# Seasonal and Inter-Annual Variations in Carbon Dioxide Exchange over an Alpine Grassland in the Eastern Qinghai-Tibetan Plateau

**DOI:** 10.1371/journal.pone.0166837

**Published:** 2016-11-18

**Authors:** Lunyu Shang, Yu Zhang, Shihua Lyu, Shaoying Wang

**Affiliations:** 1 Key Laboratory of Land Surface Process and Climate Change in Cold and Arid Regions, Northwest Institute of Eco-Environment and Resources, Chinese Academy of Sciences, Lanzhou, China; 2 College of Atmospheric Science, Chengdu University of Information Technology, Chengdu, China; Nanjing University, CHINA

## Abstract

This work analyzed carbon dioxide exchange and its controlling factors over an alpine grassland on the eastern Qinghai-Tibetan Plateau. The main results show that air temperature and photosynthetically active radiation are two dominant factors controlling daily gross primary production. Soil temperature and soil water content are the main factors controlling ecosystem respiration. Canopy photosynthetic activity is also responsible for the variation of daily ecosystem respiration other than environmental factors. No clear correlation between net ecosystem exchange and environmental factors was observed at daily scale. Temperature sensitive coefficient was observed to increase with larger soil water content. High values of temperature sensitive coefficient occurred during the periods when soil water content was high and grass was active. Annual integrated net ecosystem exchange, gross primary production and ecosystem respiration were -191, 1145 and 954 g C m^-2^ for 2010, and -250, 975 and 725 g C m^-2^ for 2011, respectively. Thus, this alpine grassland was a moderate carbon sink in both of the two years. Compared to alpine grasslands on the Qinghai-Tibetan Plateau, this alpine grassland demonstrated a much greater potential for carbon sequestration than others. Annual precipitation is a dominant factor controlling the variation of annual net ecosystem exchange over this grassland. The difference in gross primary production between the two years was not caused by the variation in annual precipitation. Instead, air temperature and the length of growing season had an important impact on annual gross primary production. Variation of annual ecosystem respiration was closely related to annual gross primary production and soil water content during the growing season.

## Introduction

Terrestrial ecosystems play a crucial role in global carbon balance. Grasslands, as an important part of terrestrial ecosystems, comprise 32% of Earth’s natural vegetation [1]. Estimated relative amounts of carbon storage in different vegetation types indicate that grassland ecosystems store possibly more than 10% of the total carbon in the biosphere [2]. It can be found that 98% of the carbon store for grassland ecosystems is sequestered below ground [3]. Grassland ecosystems, due to their belowground carbon stores, seasonal burning and regrowth, are the major players in global carbon cycle [4]. Recently, a great number of studies on carbon exchange have been conducted for different grasslands. However, it is imperative to address more aspects in order to model terrestrial carbon balance with confidence [5]. For example, we need to estimate the response of carbon balance to present climate change for various types of grasslands.

The Qinghai-Tibetan Plateau, with an area of about 2.5 million km^2^ and an average elevation of more than 4,000 m, is the largest and highest plateau on the Earth, and an important component of global terrestrial ecosystems. Major types of vegetation distributed in the Qinghai-Tibetan Plateau are alpine cold meadow, cold steppe and cold swamp meadow [6]. Several recent studies have investigated the carbon exchange and its environmental controlling factors in the alpine grasslands on the Qinghai-Tibetan Plateau [7–13]. These studies showed that most of the alpine grasslands are important carbon sinks [8–10, 12, 13]. Nevertheless, there was also a study carried out on the Qinghai-Tibetan Plateau indicated that the alpine meadow was a carbon source [11]. A study conducted at a higher elevation meadow indicated that the alpine grassland could be a carbon sink or source depending on the variation of precipitation pattern [7]. These studies promote our understanding of the carbon dynamics of the alpine grassland ecosystems on the Qinghai-Tibetan Plateau. However, the spatial distribution of the carbon budget and its response to climate change are still uncertain [11, 13]. Over the past several decades, the Qinghai-Tibetan Plateau has experienced evident climate warming and moistening [14–16], which have resulted in changes in atmospheric circulation and hydrological cycle and reshaped the local environment [16]. The variation of vegetation, especially grasslands, on the Qinghai-Tibetan Plateau is significantly correlated with climate change [17]. An improved understanding of the response of carbon dynamics of alpine grassland ecosystems to the changing environmental condition on the Qinghai-Tibetan Plateau is needed.

This paper investigated the carbon dioxide flux observed during a 2-year period using the eddy covariance method over an alpine grassland on the eastern Qinghai-Tibetan Plateau. The main objectives of this study are to identify environmental controlling factors for net ecosystem exchange (NEE), gross primary production (GPP) and ecosystem respiration (*R*_eco_) of an alpine grassland at daily and yearly time-scale, and quantify the seasonal and inter-annual variations of NEE, GPP and *R*_eco_.

## Materials and Methods

### Site description

The measurement site is one of the sites of Zoige Plateau Wetlands Ecosystem Research Station. It is located at an alpine meadow (33.89° N latitude, 102.14° E longitude, and 3,423 m above sea level) on the eastern Qinghai-Tibet Plateau. No specific permits were required for the described field studies. The location is not privately owned or protected, and the field studies did not involve endangered or protected species. The dominant species of the grassland are Cyperaceae and Gramineae with an average height of about 0.2 m in the growing season. The grassland is a typical alpine meadow used for sheep and yak grazing throughout the year. The ground surface at the measurement site is flat and homogenous, with the slope less than 3%. The soil of the grassland is silt clay loam. It contains 66.7% silt, 29.8% sand and 3.5% clay in the top 40 cm. Based on climate data measured at a meteorological station (34° N latitude, 102.08° E longitude, and 3,471 m above sea level) located approximately 14 km north of the study site, the multi-year average of annual air temperature is 1.9°C and the annual mean precipitation is 593 mm with most of the precipitation occurring between May and September. More details on the site have been reported in the previous documents [18, 19].

### Flux measurement

An eddy covariance (EC) system was used to continuously measure the flux of CO_2_. The EC system was mounted 3.15 m above the soil surface. It consists of a 3D sonic anemometer (CSAT-3, Campbell Scientific, Inc., Logan, UT, USA) and an open path and fast response infrared gas analyzer (LI-7500, LI-COR Biosciences Inc., Lincoln, NE, USA). The separation distance between the two sensors was 0.15 m. An air temperature and relative humidity sensor (HMP-45C, Vaisala, Helsinki, Finland) was also installed at the same height, which was used for correction of flux measurements for density effects due to heat and water vapor transfer (only the mean temperature and humidity from the slow sensor can be used in the correction). Signals from EC instrumentation were recorded at the rate of 10 Hz, and the raw data were stored in a CR3000 data logger (Campbell Scientific, Inc.). The dominant prevailing winds at the site are easterlies and southeasterlies in the summer and northwesterlies in the winter. The fetch is greater than 1.5 km for all directions at the site. Footprint analysis using a model [20] indicated that the peak for the flux footprint was approximately 58 m upwind of the system, with 90% cumulative flux footprint extending to approximately 168 m upwind (the values are calculated using the average of the measurements in 2010).

In order to assess the accuracy of the EC measurements, linear regression analysis between the sum of sensitive heat flux (H) and latent heat flux (*LE*) versus available energy (net radiation (*R*_n_) minus ground heat flux (G0)) was conducted. During 2010, the intercept, slope and coefficient of determination (*r*^2^) were 10.1 W m^-2^, 0.80, and 0.89, respectively. In 2011, we attained a relatively low closure degree of the surface energy balance. The intercept, slope and coefficient of determination (*r*^2^) were 5.2 W m^-2^, 0.77, and 0.79 respectively in 2011. However, the closure degrees for the two years were close to the mean value for 50 site-years (0.79 ± 0.01) across 22 sites in FLUXNET [21].

### Meteorology and soil measurements

Meteorological and soil variables were also measured continuously with an array of sensors. Net radiation flux and photosynthetic photon flux density were measured at 1.5 m height with a four-component net radiometer (CNR-1, Kipp and Zonen, Delft, Netherlands) and a quantum sensor (LI-190Sb, LI-COR Biosciences Inc., Lincoln, NE, USA), respectively. Precipitation was measured with a weighing gauge (T200B, Geonor, Norway) at 2 m height. Soil temperature was measured at 1, 3, 5, 10 cm depths and other deeper layers with CS107 temperature probes (Campbell Scientific, Inc.). Volumetric soil water content was measured at 5 and 10 cm depths and other deeper layers with CS616 Time Domain Reflectometer (TDR) probes (Campbell Scientific, Inc.). Soil heat flux was measured using heat flux plates (HPF01, Wohlwend Engineering, Sennwald, Switzerland) buried at 2 and 7 cm below the soil surface and deeper depths. Signals from meteorological and soil sensors were recorded as half-hourly averages with a CR23XTD data logger (Campbell Scientific, Inc.).

### Data processing and gap filling

The flux data were calculated off-line using the EddyPro software [22]. The raw data were processed to obtain 30-min averages. The main procedures included spike detection and removal [23], double coordinate rotation [24], sonic air temperature correction [25], frequency response correction [26] and correction for the effect of air density fluctuations [27].

Missing data is unavoidable and universal in continuous field measurements due to instrument malfunction or power failure. For measurements at the current site, missing data were less than 1% in 2010. However, in 2011, 12.7% of the raw data were missed due to the power failure from May 3 to 9 and from October 20 to December 1. In addition, data quality assurance criteria may generate additional gaps in the data sets due to the rejection of unreasonable and/or contaminated data. Measurements taken under the following conditions were rejected: (a) rain events, (b) low quality checked with an overall flag system [28], (c) low turbulent mixing (friction velocity < 0.1 m s^-1^) during nighttime, and (d) negative CO_2_ fluxes during the non-growing season. The effects of the above four rejection criteria on data coverage are presented in Table 1. After applying the rejection criteria, data coverage of CO_2_ fluxes is 57.9% in 2010 and 48.8% in 2011.

**Table 1:** Percentages of missing or rejected CO_2_ flux data for the two years. Data rejection criteria are: (a) rain events, (b) low quality check, (c) low turbulent mixing during nighttime, and (d) negative CO_2_ flux during non-growing season.

	Missing data	Rejected data				Total
		a	b	c	d	
2010	0.1	6.1	14.9	22.2	17.0	42.1
2011	12.7	6.2	21.4	14.2	14.3	51.2

In order to obtain the information on the daily and annual carbon flux data, a gap-filling strategy [29] was adopted to fill in missing and rejected data. The missing data caused by power failure in 2011 were filled via the look-up tables method [30]. For small gaps (< 1 hour), interpolation method was used. Large gaps of daytime missing CO_2_ flux data during the growing season were filled by a light-response function [30]:

$$F_{\text{c}}=\frac{F_{\max}\alpha Q_{\text{p}}}{\alpha Q_{\text{p}}+F_{\max}}+R_{\text{eco}}$$ (1)

where *F*_c_ (μmol m^-2 s-1^) is the net flux density of CO_2_, *F*_max_ (μmol m^-2 s-1^) the maximum CO_2_ flux at infinite light, *α* the apparent quantum yield, *Q*_p_ (μmol m^-2 s-1^) the incident photosynthetically active radiation, and *R*_eco_ the respiration from soil and plants.

Nighttime missing data during the growing season and all missing data during the non-growing season were filled using the exponential relationship [29] between the CO_2_ flux during the periods of high turbulent mixing (friction velocity > 0.1 m s^-1^) and soil temperature at the depth of 5cm:

$$F_{\text{c}}=b_{0}\exp(bT_{\text{s}})$$ (2)

where *b*_0_ and *b* are the two empirical coefficients, *T*_s_ the soil temperature, from which respiration temperature coefficient (*Q*_10_) can be estimated as

$$Q_{10}=\exp(10b)$$ (3)

The exponential relationship was also used to estimate daytime *R*_eco_. GPP was estimated by subtracting *R*_eco_ from NEE. The respiration temperature coefficient (*Q*_10_) was evaluated using 5-daytime sliding windows. CO_2_ storage term was corrected before gap filling to avoid double counting based on the one point CO_2_ concentrations from the open-path IRGA of the eddy covariance system [31].

## Results and Discussion

### Weather conditions

**Fig 1:**
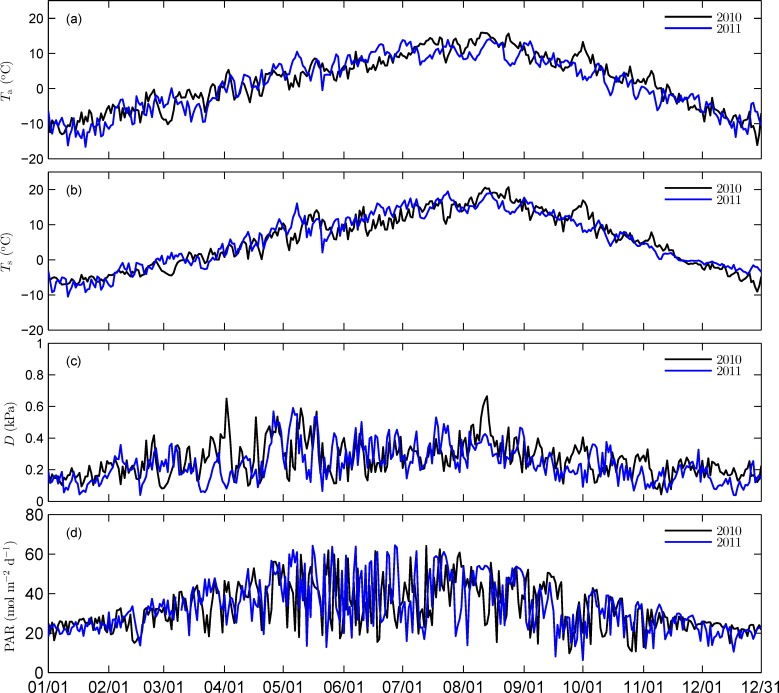
Seasonal variations of major environmental variables over the two years. (a) Daily averaged air temperature (*T_a_*), (b) daily averaged soil temperature (*T_s_*), (c) daily averaged vapor pressure deficit (*D*), and (d) daily integrated photosynthetically active radiation (PAR).

Annual mean air temperature was 3.3°C in 2010 and 2.6°C in 2011. Annual precipitation was 562.4 mm in 2010 and 637.6 mm in 2011. Based on the phonological phases of plants, each year was divided into three different periods: the pre-growing period (January 1 to April 6 in 2010 and January 1 to April 17 in 2011), the growing season (April 7 to October 31 in 2010 and April 18 to October 28 in 2011) and the post-growing period (November 1 to December 31 in 2010 and October 29 to December 31 in 2011). Daily averaged air temperature (*T*_a_, 3.15 m height), soil temperature (*T*_s_, 5 mm depth), vapor pressure deficit (*D*, 3.15 m height) and daily integrated photosynthetically active radiation (PAR) are shown in Fig 1. Averaged values of these variables for different periods of the two years are also listed in Table 2.

**Table 2:** Components of ecosystem carbon exchange and controlling environmental variables at different periods of the two years.

Period	Pre-growing		Growing		Post-growing		Annual	
	2010	2011	2010	2011	2010	2011	2010	2011
*T*_a_	-4.2	-5.8	7.6	7.6	-8.0	-5.2	3.3	2.6
*T*_s_	-1.4	-2	11.4	12.0	-3.7	-0.3	5.5	5.8
PPT	18.0	47.5	540.3	573.1	4.1	17.0	562.4	637.6
*θ*_v_	0.15	0.15	0.34	0.4	0.17	0.27	0.26	0.31
*D*	0.23	0.18	0.3	0.29	0.18	0.15	0.26	0.24
PAR	28.4	29.9	37.5	38.6	24.0	22.6	32.9	33.3
NEE	59	50	-291	-339	41	39	-191	-250
GPP	–	–	1145	975	–	–	1145	975
*R*_eco_	59	50	854	636	41	39	954	725

*T*_a_, air temperature, (^o^C); *T*_s_, soil temperature (^o^C); PPT, precipitation (mm); *θ*_v_, volumetric soil water content (m^3^ m^-3^); *D*, vapor pressure deficit (kPa); PAR, photosynthetically active radiation (mol m^-2^); NEE, net ecosystem exchange (g C m^-2^); GPP, gross primary production (g C m^-2^); and *R*_eco_, ecosystem respiration (g C m^-2^).

Air temperature during the pre-growing season in 2010 was generally higher than that in 2011. During the growing season, *T*_a_ was comparable in the two years. Daily maximum *T*_a_ reached up to 17.1°C in 2010, which was comparable to that of 15.3°C in 2011. During the post-growing season, *T*_a_ in 2010 was lower than in 2011. Variations of soil temperature followed those of air temperature during the non-growing season. However, during the growing season, the average *T*_s_ in 2010 was slightly lower than that in 2011. Vapor pressure deficit during the non-growing season in 2010 was higher than that in 2011. PAR during different periods had no remarkable differences between the two years.

**Fig 2:**
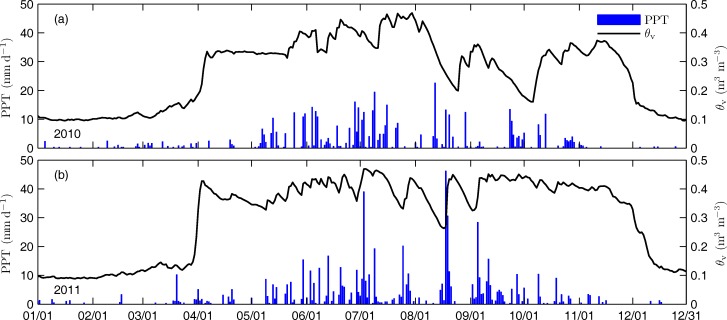
Seasonal variations of daily total precipitation (PPT) and daily averaged volumetric soil water content (*θ_v_*) over the two years. (a) For 2010 and (b) for 2011.

Daily averaged volumetric soil water content (*θ*_v_, 5 cm depth) and daily total precipitation (PPT) are shown in Fig 2. Total precipitation during the growing season in 2010 and 2011 were 540.3 and 573.1 mm, which accounted for 96% and 90% of annual precipitation, respectively. The daily maximum PPT reached up to 25 and 47 mm in 2010 and 2011, respectively. In the growing season of 2010, *θ*_v_ was generally within the range of 0.30 to 0.46 m^3^ m^-3^ except during the two dry periods when little rainfall occurred, i.e. early August and mid-September. During the dry periods, soil water content declined remarkably. *θ*_v_ reached as low as 0.19 m^-3^m^-3^ in early August and 0.16 m^3^ m^-3^ in mid-September, respectively, resulting in severe moisture stress in the growing season of 2010. In the growing season of 2011, *θ*_v_ was within the range of 0.34 to 0.47 m^3^ m^-3^ except during three relatively dry periods, i.e. mid-July, early August and late August. However, the daily minimum *θ*_v_ was not lower than 0.25 m^3^ m^-3^ during the dry periods, thereby there was no moisture stress in the growing season of 2011.

### GPP, *R*_eco_ and NEE in relation to environmental variables

**Fig 3:**
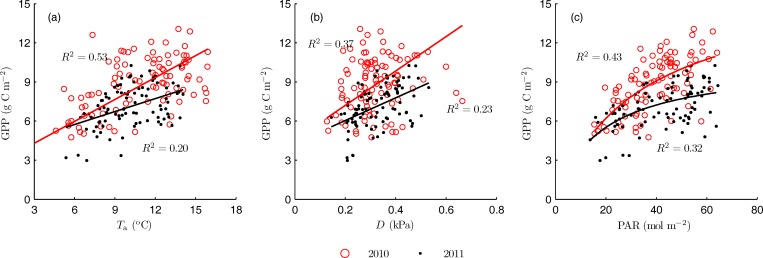
**Responses of daily gross primary production (GPP) to (a) daily averaged air temperature (*T*_a_), (b) daily averaged vapor pressure deficit (*D*), and (c) daily integrated photosynthetically active radiation (PAR) during the peak growth periods.**

GPP and *R*_eco_ are simultaneously affected by the environmental variables. Fig 3 shows the response of daily GPP to daily averaged *T*_a_, *D* and daily integrated PAR during the peak growth period (June to August). GPP was positively correlated with *T*_a_, *D* and PAR. Changes in *T*_a_ accounted for 53% (RMSE = 1.43 g C m^-2^) and 20% (RMSE = 1.41 g C m^-2^) of variability in GPP during 2010 and 2011 peak growth periods, respectively. Linear regression of GPP with *D* explained only 37% (RMSE = 1.65 g C m^-2^) and 23% (RMSE = 1.38 g C m^-2^) of the variability in GPP during the two periods, respectively. The relatively low GPP in 2011 peak growth period corresponded to lower air temperature and vapor pressure deficit during the period. Light response function of GPP to PAR explained 43% (RMSE = 1.57 g C m^-2^) and 32% (RMSE = 1.30 g C m^-2^) of the variability in GPP during the two periods respectively, indicating that the photosynthesis activity in 2010 was higher than that in 2011. The relatively low air temperature and vapor deficit in 2011 restrained the canopy photosynthesis activity.

Multiple regression of GPP with *T*_a_ and PAR provided a better fit to the data (data not shown). Changes in *T*_a_ and PAR together accounted for 71% (RMSE = 1.12 g C m^-2^) and 44% (RMSE = 1.17 g C m^-2^) of the variability in GPP during the two periods, respectively. Regression of GPP on other combinations of environmental variables did not provide a better fit to the data (data not shown). The above results suggest that the air temperature and photosynthetically active radiation are two significant environmental factors controlling the GPP at daily scale for this alpine grassland.

**Fig 4:**
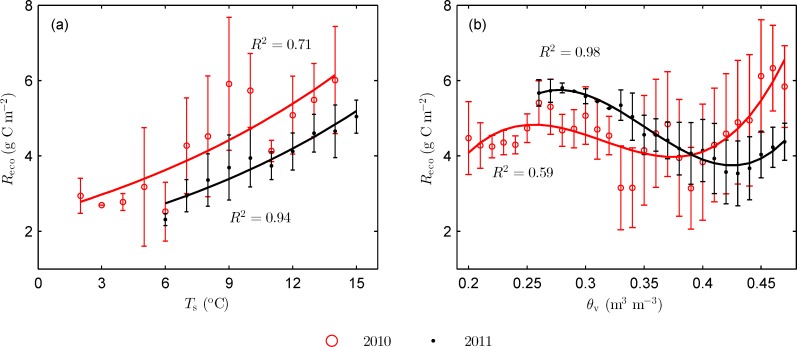
**Responses of integrated nighttime ecosystem respiration (*R*_eco_) to (a) nighttime averaged soil temperature (*T*_s_), and (b) nighttime averaged volumetric soil water content (*θ*_v_) during the peak growth periods.** *R*_eco_ data were averaged with *T*_s_ bins of 1°C and *θ*_v_ bins of 0.01 m^3^ m^-3^. Bars indicated standard errors.

Fig 4 shows response of integrated nighttime *R*_eco_ to *T*_s_ and *θ*_v_ during the peak growth period. *R*_eco_ data were averaged with *T*_s_ bins of 1°C and *θ*_v_ bins of 0.01 m^3^ m^-3^. *R*_eco_ was positively correlated with soil temperature. Changes in *T*_s_ accounted for 71% (RMSE = 0.69 g C m^-2^) and 92% (RMSE = 0.22 g C m^-2^) of the variability in *R*_eco_ during 2010 and 2011 peak growth periods, respectively. Evaluated *Q*_10_, was 1.93 and 2.03 for peak growth periods of 2010 and 2011, respectively. The *Q*_10_ values are within the range of (1.3 to 3.3) reported in a review of soil respiration study and close to the mean value [32], and also within the range of values reported in alpine grassland studies of Qinghai-Tibetan Plateau [7, 8, 10, 13]. At 30-min scale and 5-daytime window, evaluated *Q*_10_ varied from 1.27 to 4.69 for 2010 growing season and from 1.30 to 4.48 for 2011 growing season.

*R*_eco_ and *θ*_v_ showed a cubic relationship during the peak growth periods (Fig 4B). Changes in *θ*_*v*_ accounted for 59% (RMSE = 0.50 g C m^-2^) and 98% (RMSE = 0.12 g C m^-2^) of the variability in *R*_eco_ during 2010 and 2011 peak growth periods, respectively. In 2010 (2011), with the increase in *θ*_v_, *R*_eco_ showed an increasing trend when *θ*_v_ was lower than 0.26 m^3^ m^-3^ (0.28 m^3^ m^-3^) and greater than 0.39 m^3^ m^-3^ (0.43 m^3^ m^-3^), and a decreasing trend when *θ*_v_ was within the range of 0.26 to 0.39 m^3^ m^-3^ (0.28 to 0.43 m^3^ m^-3^). The positive correlation between *R*_eco_ and *θ*_v_ under high soil moisture condition occurred when grass was very active. In general, medium soil moisture condition can promote ecosystem respiration compared to low or high soil moisture conditions, which may limit ecosystem respiration. For example, Xu et al. [33] reported that soil moisture limited ecosystem respiration when soil water content is below a threshold of 0.15 m^3^ m^-3^ over a Mediterranean grassland in California. Yang et al. [34] found that ecosystem respiration and soil water content showed a quadratic relationship with the maximum *R*_eco_ occurring at the medium soil water content of 0.15 m^3^ m^-3^ over a temperate desert steppe in Inner Mongolia.

An increase in *Q*_10_ was observed in response to increase in soil water content. High *Q*_10_ values occurred during the period when soil water content was high and grass was active. High temperature sensitivity may be caused by the direct effects of temperature on the activities of plant root and microbe and indirect effects related to photosynthetic assimilation and carbon allocation to roots [29, 35]. This response had also been found on the research for ecosystem respiration in alpine grassland ecosystems in the Qinghai-Tibetan Plateau [7, 13], and in other grasslands such as the Mediterranean annual grassland [29] and northern temperate grassland [36].

**Fig 5:**
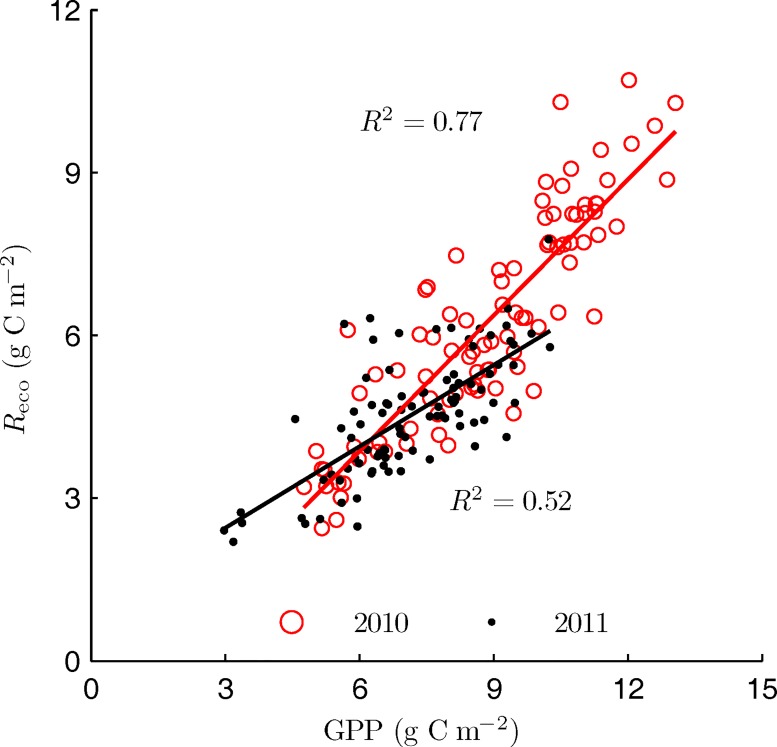
The linear relation between daily ecosystem respiration (*R*_eco_) and gross primary production (GPP) during the peak growth periods.

Although *Q*_10_ was close in the two years, *R*_eco_ in 2010 was higher than that in 2011 at the same temperature. It was found that *R*_eco_ was highly linearly correlated with GPP (Fig 5). 77% (RMSE = 0.98 g C m^-2^)and 52% (RMSE = 0.75 g C m^-2^)of the variability in *R*_eco_ could be explained by the changes in GPP during 2010 and 2011 peak growth periods, respectively. Similar results were also reported in previous studies [29], indicating that *R*_eco_ was closely related to canopy photosynthetic activity in addition to environmental factors.

There were no clear correlations between NEE and *T*_a_, *D*, PAR and *θ*_v_ at daily scale. Also no clear correlation between GPP and *θ*_v_ was observed at daily scale. However, at 30-min scale, NEE was found to be positively correlated with *T*_a_ and *D* when *T*_a_ was lower than 16.5°C and *D* was lower than 0.55 kPa. NEE was negatively correlated with *T*_a_ and *D* when *T*_a_ and *D* were higher than the above two values [18]. NEE and *θ*_v_ showed a quadratic relationship when *θ*_v_ was lower than 0.33 m^3^ m^-3^ and the maximum NEE occurred when *θ*_v_ was 0.25 m^3^ m^-3^. When *θ*_v_ was higher than 0.33 m^3^ m^-3^, NEE was positively correlated with *θ*_v_.

### Seasonal variations in NEE, GPP and *R*_eco_

**Fig 6:**
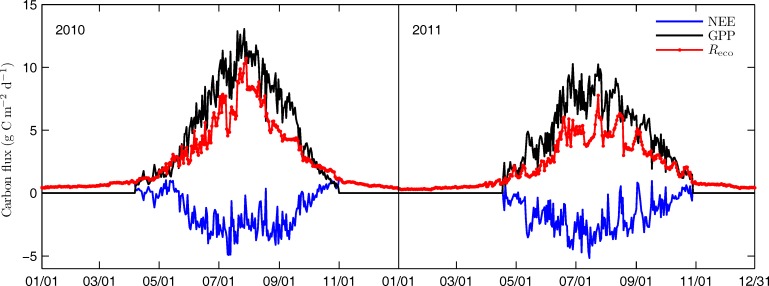
Seasonal variations of daily net ecosystem exchange (NEE), gross primary production (GPP) and ecosystem respiration (*R*_eco_) in the two years.

Seasonal variations in daily NEE, GPP and *R*_eco_ are shown in Fig 6. *R*_eco_ was less than 2 g C m^-2^ d^-1^ during nun-growing seasons of the two years. Accordingly, NEE was positive during the non-growing seasons. During the growing seasons, GPP, *R*_eco_ and NEE showed different seasonal variations between the two years. GPP and *R*_eco_ began to increase gradually since the growing seasons started. When the increment of photosynthesis exceeded that of respiration, NEE was negative. With the start of rainy season, both GPP and *R*_eco_ increased phenomenally from early May to early July in 2010. However, GPP showed a higher increasing rate than *R*_eco_, leading to a sharp increase in NEE. The daily maximum NEE reached up to -4.9 g C m^-2^ d^-1^ during early July in 2010. As *T*_a_ and *T*_s_ decreased in early July, both GPP and *R*_eco_ decreased. With the rise of temperature, GPP and *R*_eco_ then increased again. Canopy photosynthesis and ecosystem respiration peaked in late July. The daily maximum GPP and *R*_eco_ reached up to 12.9 and 10.7 g C m^-2^ d^-1^ respectively in 2010.

In 2011, the daily maximum NEE reached up to 5.2 g C m^-2^ d^-1^ during mid-July. GPP showed a sharp increase in early May due to the rapidly rising air temperature and appropriate soil moisture and then decreased in mid-May. With the rising temperature and increasing rainfall from late May, GPP showed a rapid increase and peaked in late June. The daily maximum GPP reached up to 10.3 g C m^-2^ d^-1^ in late June. GPP then decreased in early July and late July, and increased in mid-July and early August. The daily maximum GPP in mid-July (10.2 g C m^-2^ d^-1^) almost reached up to that in late June. *R*_eco_ increased from Mid-May to Mid-June, and fluctuated till early July. *R*_eco_ then increased again and reached its peak in mid-July, with the daily maximum *R*_eco_ reached up to 7.8 g C m^-2^ d^-1^. Both GPP and *R*_eco_ decreased during the late growing seasons in both years.

Seasonal variations in GPP were consistent with the changes in air temperature in both of the two growing seasons. Changes in *T*_a_ during early June to mid-July were similar in both seasons. Changes in GPP displayed a similar pattern. However, in late July, *T*_a_ showed different variations between the two seasons. Both *T*_a_ and GPP increased and reached their peaks in late July in 2010. In 2011, however, both of them decreased during late July. The decrease in air temperature during late July in 2011 prevented GPP from increasing further, resulting in as large as 2.6 g C m^-2^ d^-1^ of difference in daily maximum GPP between the two years.

*R*_eco_ in 2010 growing season ranged from 0.8 to 10.7 g C m^-2^ d^-1^, as compared to that of 0.8 to 7.8 g C m^-2^ d^-1^ during 2011 growing season. The difference in *R*_eco_ between the two seasons might be caused by the seasonal differences in both soil temperature and soil moisture. *T*_s_ showed an increasing trend with a rise from 9.3 to 13.9°C during late July in 2010 growing season, whereas *T*_s_ decreased from 17.1 to 12.1 ^o^C in 2011. In 2010, *θ*_v_ was greater than 0.42 m^3^ m^-3^ during the period when the respiration rate was high (Figs 2A and 4B). In contrast, in 2011, *θ*_v_ was within the range of 0.33 to 0.42 m^3^ m^-3^ during the period when respiration was relatively low (Figs 2B and 4B). As a result, *R*_eco_ increased during late July in the 2010 growing season but decreased during late July in the 2011 growing season. The divergence led to as much as 2.9 g C m^-2^ d^-1^ of difference in daily maximum *R*_eco_ between the two years. *R*_eco_ showed sharp increases during three dry periods in the growing season of 2011, resulting in remarkable decreases in NEE. The observation demonstrated that changes in soil moisture played an important role during the process.

### Cumulative NEE, GPP and *R*_eco_

**Fig 7:**
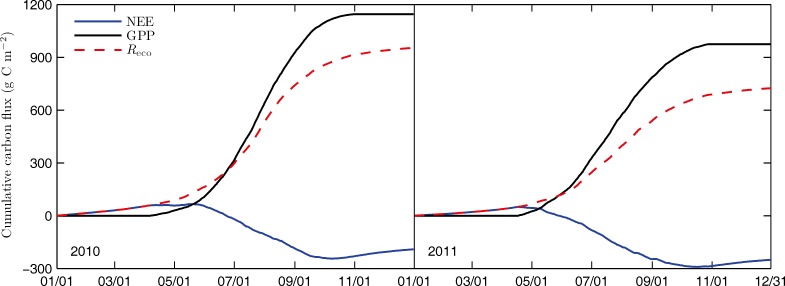
Cumulative net ecosystem exchange (NEE), gross primary production (GPP) and ecosystem respiration (*R*_eco_) over the two years.

The cumulative NEE, GPP and *R*_eco_ over the two years are shown in Fig 7. Cumulative NEE, GPP and *R*_eco_ were -191, 1145 and 954 g C m^-2 ^for 2010, and -250, 975 and 725 g C m^-2^ for 2011, respectively. According to the cumulative NEE data, this alpine grassland was a moderate carbon sink in both of the two years. Annual integrated values of NEE of alpine grasslands on the Qinghai-Tibetan Plateau reported in previous studies were within the range of 173 to -193 g C m^−2^ yr^−1^ [7, 8, 10, 11]. Compared to alpine grasslands on the Qinghai-Tibetan Plateau, this alpine grassland showed a much greater potential for carbon sequestration than others.

The ecosystem gained more carbon in 2010 growing season than that in 2011 growing season via photosynthesis, and produced more CO_2_ in 2010 than that in 2011 via respiration regardless in growing season or non-growing season (Table 2). However, the system fixed less carbon in 2010 than that in 2011. It is highly probable that the annual precipitation is a dominant variable controlling the NEE for this grassland. The annual precipitation in 2011 was 13.4% more than that in 2010, and the ecosystem fixed 30.9% more carbon in 2011 than that in 2010.

Many research found that annual grassland productivity is positively correlated with annual precipitation [37, 38]. This study, in contrast, found that the difference in GPP between the two years was not caused by the variation in annual precipitation. Though much more precipitation happened in 2011, GPP was 170 g C m^-2^ less than that in 2010. For present alpine grassland, air temperature had more influence than precipitation in determining the GPP, as described in previous sections. In addition, the lengths of growing seasons also had an important impact on GPP [29]. The length of growing season in 2011 was 14 days shorter than that in 2010, which may be a reason why GPP was lower in 2011. Cumulative *R*_eco_ was 83% and 74% of GPP in 2010 and 2011, respectively. Lower *R*_eco_ in 2011 was mainly caused by lower GPP and corresponding soil water content during the growing season. Despite the decrease of GPP and *R*_eco_ in 2011 compared with the previous year, *R*_eco_ reduced much more than GPP, resulting in more NEE in 2011.

## Conclusions

Alpine grasslands comprise most of the natural vegetation in the Qinghai-Tibetan Plateau. Over the past several decades, the Qinghai-Tibetan Plateau experienced evident climate warming and moistening. Understanding carbon dynamics of alpine grassland ecosystems on the Qinghai-Tibetan Plateau in response to changing environmental conditions is critical to accurately model carbon balance. Carbon dioxide exchange over the alpine grassland on the eastern Qinghai-Tibetan Plateau was measured using the eddy covariance method. The main results show that air temperature and photosynthetically active radiation are dominant factors controlling the GPP at daily scale. Soil temperature and soil water content are main variables controlling *R*_eco_. Canopy photosynthetic activity is also responsible for the variation of daily *R*_eco_ other than environmental factors. No clear correlation between net ecosystem exchange and environmental factors was observed at daily scale. Temperature sensitive coefficient *Q*_10_ was observed to increase with larger soil water content. High *Q*_10_ values occurred during the periods when soil water content was high and grass was active. Annual integrated NEE, GPP and *R*_eco_ were -191, 1145 and 954 g C m^-2^ for 2010, and -250, 975 and 725 g C m^-2^ for 2011, respectively. According to the annual NEE data, this alpine grassland was a moderate carbon sink in both of the two years. Compared to alpine grasslands on the Qinghai-Tibetan Plateau, this alpine grassland showed a much greater potential for carbon sequestration. Annual precipitation was a dominant variable controlling the variation of annual NEE of this grassland. The difference in GPP between the two years was not caused by the variation in annual precipitation. Instead, air temperature had more influences than precipitation in determining the GPP. In addition, the length of growing season also had an important impact on annual GPP. The variation of annual *R*_eco_ was closely related to annual GPP and soil water content during the growing seasons. Under the background of global climate change, more studies are needed to understand how carbon dynamics of alpine grassland ecosystems in the Qinghai-Tibetan Plateau respond to changing environmental conditions.

## Supporting Information

S1 File Data set of the Components of ecosystem carbon exchange and controlling environmental variables. *T*_a_, air temperature, (°C); *T*_s_, soil temperature (°C); PPT, precipitation (mm); *θ*_v_, volumetric soil water content (m^3^ m^-3^); *D*, vapor pressure deficit (kPa); PAR, photosynthetically active radiation (mol m^-2^); NEE, net ecosystem exchange (g C m^-2^); GPP, gross primary production (g C m^-2^); and *R*_eco_, ecosystem respiration (g C m^-2^).
(PDF)
