# Supplementary material for: Seasonal and Inter-Annual Variations in Carbon Dioxide Exchange over an Alpine Grassland in the Eastern Qinghai-Tibetan Plateau
Source: PLoS One. 2016 Nov 18;11(11):e0166837. doi: 10.1371/journal.pone.0166837 (PMC5115830; doi:10.1371/journal.pone.0166837)
Supplement: S1 File — Ta, air temperature, (°C); Ts, soil temperature (°C); PPT, precipitation (mm); θv, volumetric soil water content (m3 m-3); D, vapor pressure deficit (kPa); PAR, photosynthetically active radiation (mol m-2); NEE, net ecosystem exchange (g C m-2); GPP, gross primary production (g C m-2); and Reco, ecosystem respiration (g C m-2). (PDF) [file pone.0166837.s001.pdf]

Date	Ta	Ts	D	PAR	PPT	θv	NEE	GPP	Reco
2010-1-1	-9.5	-4.4	0.182	25.01	0	0.111	0.440	0.000	0.440
2010-1-2	-12.5	-5.7	0.126	19.78	0	0.106	0.413	0.000	0.413
2010-1-3	-9.1	-5.2	0.170	25.32	0	0.106	0.425	0.000	0.425
2010-1-4	-9.9	-5.0	0.146	16.31	0	0.106	0.406	0.000	0.406
2010-1-5	-9.5	-5.2	0.148	20.28	2.3	0.105	0.482	0.000	0.482
2010-1-6	-10.7	-5.8	0.158	23.95	0	0.103	0.428	0.000	0.428
2010-1-7	-12.5	-6.4	0.123	26.05	0	0.100	0.494	0.000	0.494
2010-1-8	-13.0	-7.1	0.103	25.65	0	0.098	0.431	0.000	0.431
2010-1-9	-12.8	-7.0	0.150	22.14	0	0.097	0.467	0.000	0.467
2010-1-10	-11.4	-7.0	0.137	25.92	0.3	0.097	0.498	0.000	0.498
2010-1-11	-10.4	-6.9	0.165	20.45	0	0.097	0.488	0.000	0.488
2010-1-12	-10.7	-6.6	0.166	25.71	0	0.096	0.480	0.000	0.480
2010-1-13	-8.4	-5.5	0.211	25.83	0.3	0.099	0.481	0.000	0.481
2010-1-14	-9.7	-5.9	0.171	24.31	0	0.098	0.472	0.000	0.472
2010-1-15	-11.9	-6.9	0.149	24.81	0	0.096	0.498	0.000	0.498
2010-1-16	-9.2	-6.8	0.162	24.43	0	0.095	0.487	0.000	0.487
2010-1-17	-9.5	-6.3	0.180	26.78	0	0.096	0.491	0.000	0.491
2010-1-18	-6.8	-4.3	0.094	26.80	0	0.100	0.537	0.000	0.537
2010-1-19	-10.2	-5.6	0.120	27.31	0.3	0.098	0.550	0.000	0.550
2010-1-20	-8.0	-5.1	0.157	24.96	0	0.099	0.511	0.000	0.511
2010-1-21	-9.4	-6.2	0.157	25.17	0	0.097	0.489	0.000	0.489
2010-1-22	-6.2	-5.0	0.225	24.43	0	0.099	0.500	0.000	0.500
2010-1-23	-7.1	-4.7	0.179	27.90	0	0.100	0.493	0.000	0.493
2010-1-24	-10.9	-4.9	0.103	21.45	0	0.100	0.521	0.000	0.521
2010-1-25	-10.9	-5.8	0.160	27.36	0.3	0.098	0.504	0.000	0.504
2010-1-26	-10.4	-6.3	0.162	25.04	0	0.097	0.521	0.000	0.521
2010-1-27	-10.1	-6.2	0.160	28.06	0	0.096	0.502	0.000	0.502
2010-1-28	-7.1	-5.5	0.248	22.59	0	0.098	0.546	0.000	0.546
2010-1-29	-9.0	-6.0	0.192	28.85	0	0.096	0.519	0.000	0.519
2010-1-30	-7.3	-5.3	0.181	21.75	0	0.098	0.444	0.000	0.444
2010-1-31	-5.7	-5.0	0.254	20.31	0	0.099	0.487	0.000	0.487
2010-2-1	-5.0	-4.3	0.238	25.03	0	0.101	0.485	0.000	0.485
2010-2-2	-7.0	-4.4	0.243	19.29	0	0.102	0.515	0.000	0.515
2010-2-3	-4.6	-4.3	0.218	19.78	0	0.102	0.511	0.000	0.511
2010-2-4	-5.2	-3.8	0.270	23.04	0.3	0.103	0.509	0.000	0.509
2010-2-5	-5.5	-3.5	0.238	31.51	0	0.104	0.494	0.000	0.494
2010-2-6	-4.8	-2.9	0.208	31.56	0	0.106	0.495	0.000	0.495
2010-2-7	-9.1	-3.7	0.108	32.57	0	0.105	0.559	0.000	0.559
2010-2-8	-5.6	-3.3	0.220	25.52	0	0.105	0.567	0.000	0.567
2010-2-9	-8.3	-4.0	0.147	27.91	2.4	0.103	0.550	0.000	0.550
2010-2-10	-5.6	-3.5	0.259	21.02	0	0.104	0.540	0.000	0.540
2010-2-11	-7.1	-4.2	0.180	24.35	0	0.103	0.531	0.000	0.531
2010-2-12	-8.0	-4.8	0.206	29.37	0	0.101	0.537	0.000	0.537
2010-2-13	-5.3	-3.4	0.225	16.40	0.4	0.104	0.510	0.000	0.510
2010-2-14	-6.5	-3.8	0.205	14.99	0	0.103	0.538	0.000	0.538
2010-2-15	-6.0	-2.7	0.142	16.00	1	0.107	0.500	0.000	0.500
2010-2-16	-6.3	-3.0	0.139	18.01	0	0.107	0.514	0.000	0.514
2010-2-17	-9.7	-4.8	0.149	16.38	0.3	0.103	0.528	0.000	0.528
2010-2-18	-6.6	-4.3	0.252	21.25	0.3	0.103	0.527	0.000	0.527
2010-2-19	-6.6	-4.2	0.198	31.54	0	0.103	0.562	0.000	0.562
2010-2-20	-1.1	-2.5	0.384	32.67	0	0.107	0.530	0.000	0.530
2010-2-21	-3.7	-2.0	0.207	29.24	0	0.110	0.544	0.000	0.544
2010-2-22	-2.1	-1.6	0.241	31.59	0	0.112	0.566	0.000	0.566

2010-2-23	-1.3	-1.4	0.366	32.34	0	0.115	0.555	0.000	0.555
2010-2-24	-0.3	-0.7	0.417	22.97	0	0.119	0.539	0.000	0.539
2010-2-25	-3.3	-0.4	0.249	32.32	0.5	0.121	0.540	0.000	0.540
2010-2-26	-4.9	-1.3	0.169	32.22	1.4	0.124	0.589	0.000	0.589
2010-2-27	-6.6	-2.3	0.098	31.76	0.3	0.119	0.557	0.000	0.557
2010-2-28	-8.2	-3.2	0.081	30.43	0	0.116	0.589	0.000	0.589
2010-3-1	-8.9	-3.4	0.082	32.97	0	0.111	0.578	0.000	0.578
2010-3-2	-8.9	-3.8	0.101	26.48	1	0.109	0.657	0.000	0.657
2010-3-3	-10.2	-4.1	0.120	25.86	0	0.107	0.628	0.000	0.628
2010-3-4	-9.5	-4.4	0.154	28.65	1.7	0.105	0.629	0.000	0.629
2010-3-5	-9.0	-4.4	0.192	21.90	0.7	0.104	0.631	0.000	0.631
2010-3-6	-6.3	-3.8	0.234	39.41	1.6	0.106	0.656	0.000	0.656
2010-3-7	-2.4	-1.9	0.347	35.84	0	0.110	0.650	0.000	0.650
2010-3-8	-2.1	-0.8	0.356	26.10	0	0.113	0.615	0.000	0.615
2010-3-9	-1.0	-0.4	0.332	40.67	0	0.119	0.679	0.000	0.679
2010-3-10	-2.6	-0.1	0.169	41.00	0	0.122	0.598	0.000	0.598
2010-3-11	-2.5	-0.1	0.193	35.09	0	0.130	0.651	0.000	0.651
2010-3-12	-2.5	-0.4	0.246	37.45	2.2	0.127	0.796	0.000	0.796
2010-3-13	-0.6	0.8	0.332	30.74	0	0.130	0.801	0.000	0.801
2010-3-14	-0.3	2.1	0.347	26.62	0	0.134	0.809	0.000	0.809
2010-3-15	-1.7	1.7	0.215	34.90	0.6	0.140	0.811	0.000	0.811
2010-3-16	-3.0	0.0	0.190	39.49	0	0.146	0.837	0.000	0.837
2010-3-17	-1.5	1.4	0.274	42.32	0	0.144	0.817	0.000	0.817
2010-3-18	-2.3	1.0	0.131	44.11	0.3	0.148	0.816	0.000	0.816
2010-3-19	-2.7	1.7	0.139	36.00	0	0.151	0.847	0.000	0.847
2010-3-20	-1.2	1.6	0.224	37.48	0	0.153	0.818	0.000	0.818
2010-3-21	-2.8	0.4	0.330	30.83	0.8	0.157	0.851	0.000	0.851
2010-3-22	-6.7	-1.0	0.245	20.27	0	0.151	0.802	0.000	0.802
2010-3-23	-4.5	-0.5	0.272	29.01	0.4	0.140	0.823	0.000	0.823
2010-3-24	-2.0	1.6	0.287	18.37	0.8	0.138	0.763	0.000	0.763
2010-3-25	0.3	3.1	0.408	30.93	0	0.145	0.787	0.000	0.787
2010-3-26	1.3	1.5	0.397	37.91	0	0.152	0.835	0.000	0.835
2010-3-27	0.8	2.0	0.357	47.40	0	0.161	0.811	0.000	0.811
2010-3-28	-3.2	0.0	0.294	40.17	1.5	0.169	0.859	0.000	0.859
2010-3-29	-3.4	0.0	0.243	40.97	0.3	0.161	0.901	0.000	0.901
2010-3-30	1.2	1.9	0.425	33.92	0	0.167	0.845	0.000	0.845
2010-3-31	0.3	2.1	0.400	20.22	1.6	0.178	0.755	0.000	0.755
2010-4-1	0.9	0.8	0.403	46.43	0	0.187	0.871	0.000	0.871
2010-4-2	4.2	3.1	0.650	40.75	0	0.197	0.890	0.000	0.890
2010-4-3	5.3	4.2	0.521	45.76	0	0.223	0.996	0.000	0.996
2010-4-4	3.3	4.2	0.383	36.61	0	0.274	0.951	0.000	0.951
2010-4-5	0.1	3.5	0.178	30.16	0	0.312	0.842	0.000	0.842
2010-4-6	-3.2	0.9	0.121	25.02	0	0.335	0.830	0.000	0.830
2010-4-7	-2.2	1.3	0.225	39.44	2.4	0.321	0.259	0.564	0.823
2010-4-8	-2.1	2.0	0.208	46.04	0	0.314	0.208	0.652	0.860
2010-4-9	-1.2	3.8	0.344	44.11	0	0.315	0.299	0.716	1.015
2010-4-10	1.8	5.0	0.312	40.70	0	0.324	0.358	0.671	1.029
2010-4-11	1.3	5.2	0.218	41.00	0	0.334	0.235	0.916	1.151
2010-4-12	-0.4	4.5	0.205	38.44	0	0.337	0.198	0.957	1.155
2010-4-13	-2.1	0.2	0.098	38.00	0	0.336	0.128	1.008	1.136
2010-4-14	-4.0	3.9	0.109	43.32	0	0.332	-0.268	1.391	1.123
2010-4-15	-0.8	4.6	0.188	46.64	0	0.334	-0.560	1.751	1.191
2010-4-16	1.6	6.0	0.257	52.77	0	0.335	0.035	1.150	1.185
2010-4-17	3.3	5.2	0.531	45.28	0	0.338	-0.043	1.226	1.183

2010-4-18	1.3	3.8	0.331	41.92	0	0.337	0.058	1.152	1.210
2010-4-19	-2.0	2.2	0.113	36.31	2.8	0.335	0.278	0.928	1.207
2010-4-20	-2.1	0.1	0.143	21.42	1.2	0.334	0.506	0.699	1.205
2010-4-21	0.2	3.4	0.293	24.28	0.7	0.327	0.280	0.754	1.033
2010-4-22	4.2	6.2	0.427	43.65	0	0.333	0.102	0.974	1.077
2010-4-23	4.1	7.0	0.452	39.34	0	0.334	0.118	0.975	1.093
2010-4-24	4.5	7.5	0.476	24.94	0	0.334	0.115	1.038	1.154
2010-4-25	3.9	6.3	0.396	31.81	0	0.334	-0.599	1.642	1.042
2010-4-26	5.2	7.7	0.441	55.97	0	0.334	-1.137	2.254	1.117
2010-4-27	4.5	8.1	0.436	56.11	0	0.336	-0.693	1.908	1.215
2010-4-28	3.8	6.8	0.535	53.45	0	0.334	-0.080	1.322	1.242
2010-4-29	3.9	9.0	0.434	44.30	0	0.333	-0.293	1.557	1.264
2010-4-30	3.4	8.6	0.406	48.07	0	0.333	-0.391	1.674	1.284
2010-5-1	4.4	10.3	0.284	54.50	0	0.333	-0.200	1.896	1.696
2010-5-2	4.8	9.5	0.306	45.66	0	0.333	0.244	1.535	1.779
2010-5-3	2.9	4.9	0.148	22.74	0.3	0.329	0.413	1.271	1.684
2010-5-4	-0.3	2.7	0.128	45.88	0	0.326	-0.190	2.052	1.862
2010-5-5	1.5	6.3	0.386	43.18	0.5	0.328	0.271	1.607	1.877
2010-5-6	0.5	5.7	0.219	40.25	1.6	0.327	0.714	1.276	1.990
2010-5-7	3.7	7.5	0.199	21.19	6.6	0.330	0.368	1.431	1.799
2010-5-8	1.8	4.6	0.514	53.79	4.6	0.328	1.022	1.303	2.325
2010-5-9	0.6	5.6	0.459	17.97	2	0.326	-0.066	1.611	1.545
2010-5-10	4.4	8.4	0.588	59.64	0	0.328	0.428	1.558	1.986
2010-5-11	4.8	10.6	0.531	47.66	0	0.329	0.166	2.186	2.352
2010-5-12	4.2	10.4	0.394	34.90	5.1	0.330	1.027	1.397	2.424
2010-5-13	3.5	10.0	0.333	29.26	10.4	0.329	0.915	1.310	2.225
2010-5-14	4.7	12.6	0.398	49.15	0	0.329	0.866	1.846	2.712
2010-5-15	6.6	12.8	0.475	53.48	5.5	0.328	0.976	2.104	3.080
2010-5-16	7.9	11.0	0.326	46.07	0.8	0.328	0.334	2.604	2.938
2010-5-17	8.6	13.4	0.450	43.41	0	0.327	0.102	2.489	2.591
2010-5-18	8.4	14.1	0.568	42.50	0	0.327	0.354	2.511	2.865
2010-5-19	6.4	13.3	0.295	41.33	0.3	0.326	0.101	2.807	2.909
2010-5-20	5.6	8.8	0.176	36.63	5	0.324	0.175	2.643	2.818
2010-5-21	7.2	11.5	0.368	18.98	0	0.313	0.149	1.984	2.133
2010-5-22	5.0	10.9	0.189	56.34	0	0.346	-1.472	3.711	2.239
2010-5-23	5.6	8.6	0.174	49.51	0	0.351	-0.233	2.997	2.764
2010-5-24	5.0	8.5	0.227	61.83	0	0.348	-0.201	3.909	3.708
2010-5-25	4.4	9.2	0.189	27.39	12.3	0.370	-0.054	2.888	2.834
2010-5-26	4.8	7.6	0.150	21.28	0	0.371	-0.661	3.038	2.378
2010-5-27	2.5	5.9	0.113	59.77	0	0.385	-1.362	4.696	3.334
2010-5-28	2.2	5.8	0.114	38.74	0	0.405	-1.695	4.713	3.019
2010-5-29	4.8	9.1	0.340	57.56	0.4	0.403	-1.641	5.253	3.612
2010-5-30	4.3	7.8	0.291	17.28	10.8	0.398	-0.158	2.857	2.698
2010-5-31	5.6	9.0	0.366	20.21	11.9	0.397	-1.825	4.155	2.330
2010-6-1	6.4	10.0	0.401	31.85	0	0.391	-2.719	5.163	2.444
2010-6-2	6.3	10.3	0.312	31.82	0.7	0.384	-2.887	5.485	2.598
2010-6-3	2.8	5.8	0.127	57.78	0	0.388	-2.260	5.982	3.722
2010-6-4	5.7	9.0	0.304	36.89	14.2	0.407	-1.558	4.760	3.202
2010-6-5	5.5	8.1	0.201	33.74	0.8	0.406	-2.381	5.650	3.269
2010-6-6	5.8	9.0	0.277	45.88	12.7	0.410	-1.064	6.000	4.937
2010-6-7	10.5	13.0	0.387	23.88	10.8	0.333	-1.630	5.166	3.536
2010-6-8	6.8	8.6	0.285	52.67	0	0.339	-2.868	7.144	4.277
2010-6-9	8.6	14.0	0.427	19.18	2.9	0.343	-2.252	5.538	3.286
2010-6-10	7.9	12.3	0.359	26.18	0	0.340	-2.575	5.585	3.010

2010-6-11	8.1	15.1	0.314	32.18	1	0.336	-2.707	6.569	3.862
2010-6-12	7.3	8.8	0.163	35.39	2.1	0.331	-2.429	6.449	4.020
2010-6-13	4.6	6.8	0.133	20.21	3.4	0.383	-2.045	5.263	3.218
2010-6-14	5.8	8.3	0.263	43.96	1.5	0.399	-3.205	7.751	4.546
2010-6-15	5.5	8.4	0.185	24.92	0	0.390	-1.934	5.884	3.950
2010-6-16	6.7	11.3	0.297	31.95	0.6	0.382	-2.551	6.403	3.852
2010-6-17	5.2	8.9	0.184	61.48	0	0.378	-2.859	8.460	5.601
2010-6-18	6.7	10.4	0.243	15.51	7.7	0.418	-1.164	5.031	3.867
2010-6-19	9.0	12.7	0.242	16.87	0.6	0.418	-1.708	5.224	3.516
2010-6-20	7.0	9.7	0.189	41.39	0.6	0.446	-4.010	7.982	3.973
2010-6-21	7.8	11.0	0.343	50.93	0	0.438	-3.225	8.149	4.925
2010-6-22	6.5	8.5	0.186	32.33	0	0.427	-3.619	7.782	4.163
2010-6-23	6.5	8.6	0.221	27.76	1.7	0.427	-3.049	7.050	4.002
2010-6-24	9.3	11.9	0.373	44.40	0	0.420	-3.310	8.629	5.319
2010-6-25	9.0	11.5	0.279	42.39	6.9	0.418	-3.340	9.313	5.973
2010-6-26	7.3	8.9	0.296	35.75	0	0.419	-1.634	8.022	6.388
2010-6-27	9.8	12.8	0.385	26.78	1	0.415	-1.334	7.347	6.013
2010-6-28	9.1	10.4	0.293	22.57	16	0.410	-1.487	6.845	5.358
2010-6-29	8.9	11.0	0.325	33.23	5.6	0.403	-3.500	8.863	5.363
2010-6-30	10.1	14.6	0.408	34.16	14	0.392	-1.681	7.639	5.958
2010-7-1	9.4	11.2	0.210	35.88	0.5	0.387	-3.309	9.632	6.323
2010-7-2	9.1	11.1	0.279	45.63	9.7	0.406	-2.538	10.199	7.661
2010-7-3	8.5	12.6	0.262	41.27	12.4	0.402	-2.889	10.563	7.674
2010-7-4	10.1	14.4	0.322	36.11	0.3	0.392	-3.076	9.500	6.424
2010-7-5	9.6	12.2	0.348	58.32	0	0.378	-3.488	11.337	7.849
2010-7-6	10.5	12.0	0.321	40.79	4	0.369	-1.919	9.120	7.201
2010-7-7	11.9	15.9	0.318	45.34	0.3	0.365	-2.809	10.430	7.621
2010-7-8	11.9	15.5	0.317	34.87	13	0.355	-3.009	10.716	7.707
2010-7-9	12.1	15.1	0.277	19.63	19.4	0.349	-3.476	8.567	5.091
2010-7-10	12.8	14.8	0.279	30.23	0	0.347	-4.882	9.445	4.564
2010-7-11	12.6	13.6	0.204	32.07	2.7	0.347	-4.923	9.899	4.977
2010-7-12	12.6	14.4	0.208	23.04	4.8	0.425	-2.746	7.582	4.836
2010-7-13	13.6	15.8	0.274	64.05	0	0.443	-4.902	11.249	6.347
2010-7-14	13.8	15.7	0.283	28.01	4.8	0.450	-0.638	7.480	6.842
2010-7-15	14.4	16.7	0.341	31.47	7.8	0.448	-2.629	9.198	6.568
2010-7-16	14.1	16.7	0.306	23.99	14.9	0.464	-2.109	8.388	6.278
2010-7-17	13.0	14.4	0.291	48.13	0	0.451	-4.024	10.443	6.419
2010-7-18	12.5	15.9	0.394	38.02	0	0.438	-3.057	8.946	5.889
2010-7-19	12.5	15.4	0.303	52.71	0.3	0.420	-3.854	10.006	6.152
2010-7-20	14.6	17.0	0.340	62.42	0	0.420	-4.007	12.877	8.869
2010-7-21	14.1	16.4	0.303	44.36	6.9	0.442	-1.784	10.537	8.754
2010-7-22	9.5	11.4	0.192	41.43	8.6	0.467	-1.979	11.400	9.421
2010-7-23	7.3	10.2	0.288	54.28	0	0.458	-2.742	12.606	9.864
2010-7-24	9.2	12.2	0.228	45.80	0	0.451	-1.664	10.732	9.068
2010-7-25	10.4	12.0	0.219	33.72	0	0.457	-2.688	11.545	8.857
2010-7-26	11.7	15.7	0.351	44.10	0.5	0.452	-2.547	12.079	9.531
2010-7-27	13.7	15.8	0.259	54.84	0	0.448	-2.772	13.059	10.286
2010-7-28	14.3	16.4	0.286	45.71	0	0.453	-0.193	10.493	10.300
2010-7-29	12.8	15.0	0.270	58.20	0	0.463	-1.318	12.024	10.706
2010-7-30	12.3	15.9	0.447	60.75	0	0.469	-2.516	10.757	8.241
2010-7-31	9.7	13.7	0.288	53.45	0	0.455	-2.101	10.337	8.236
2010-8-1	10.0	14.1	0.372	53.95	1.8	0.443	-2.851	11.287	8.436
2010-8-2	11.4	15.9	0.402	43.35	0.3	0.424	-2.624	10.846	8.222
2010-8-3	11.8	15.4	0.282	47.44	0	0.403	-2.987	11.263	8.276

2010-8-4	14.0	17.2	0.326	42.34	4.6	0.418	-1.616	10.093	8.477
2010-8-5	14.1	17.8	0.475	53.53	0	0.440	-2.634	11.042	8.407
2010-8-6	12.9	16.1	0.486	44.07	0	0.429	-2.791	11.045	8.254
2010-8-7	12.3	15.3	0.360	47.43	0	0.418	-1.994	10.156	8.162
2010-8-8	14.2	17.4	0.423	41.72	0	0.405	-3.742	11.746	8.004
2010-8-9	14.3	18.9	0.383	38.55	0	0.390	-3.298	11.011	7.712
2010-8-10	15.9	18.8	0.527	45.60	0	0.373	-2.886	11.313	8.427
2010-8-11	15.9	19.8	0.600	41.48	4	0.358	-1.343	10.174	8.832
2010-8-12	15.8	20.5	0.640	23.88	22.6	0.341	-0.694	8.161	7.467
2010-8-13	15.8	20.2	0.664	24.72	3.2	0.324	-0.639	7.529	6.890
2010-8-14	15.3	20.3	0.510	30.02	0	0.308	-2.193	9.185	6.992
2010-8-15	14.5	18.7	0.415	55.60	0	0.292	-3.359	10.700	7.340
2010-8-16	14.5	19.1	0.408	45.68	1.2	0.280	-2.215	9.451	7.236
2010-8-17	14.0	19.1	0.347	38.48	0.3	0.269	-2.520	10.236	7.716
2010-8-18	13.1	18.9	0.383	15.41	13.2	0.258	0.355	5.739	6.095
2010-8-19	11.8	17.6	0.281	51.41	0	0.247	-3.381	9.708	6.327
2010-8-20	12.8	18.7	0.368	35.29	11.5	0.238	-2.969	8.792	5.822
2010-8-21	13.1	18.2	0.356	41.81	0	0.228	-3.762	9.454	5.693
2010-8-22	11.8	17.5	0.263	25.18	3.8	0.219	-3.660	8.641	4.982
2010-8-23	14.9	20.0	0.366	28.88	0	0.211	-3.200	8.015	4.815
2010-8-24	15.7	20.7	0.468	48.12	0	0.202	-3.468	8.509	5.042
2010-8-25	13.6	17.0	0.235	46.93	0.4	0.199	-2.831	8.534	5.703
2010-8-26	13.0	16.3	0.258	42.22	0	0.288	-2.340	8.059	5.719
2010-8-27	12.6	16.2	0.398	52.25	0	0.319	-3.521	8.893	5.372
2010-8-28	12.0	17.2	0.406	53.68	0	0.313	-4.114	9.536	5.421
2010-8-29	11.4	16.5	0.317	35.77	12.4	0.297	-2.244	7.481	5.237
2010-8-30	13.2	17.1	0.276	27.64	0.6	0.288	-1.076	6.359	5.283
2010-8-31	10.8	13.9	0.183	39.15	0	0.282	-4.023	9.043	5.020
2010-9-1	11.9	17.0	0.387	37.13	0.4	0.335	-2.514	7.428	4.914
2010-9-2	9.5	14.4	0.207	48.06	0	0.334	-3.402	8.301	4.899
2010-9-3	9.5	14.6	0.346	52.02	0	0.358	-3.653	8.215	4.562
2010-9-4	7.0	11.3	0.232	17.65	0.3	0.352	-1.467	5.602	4.135
2010-9-5	7.9	12.5	0.280	24.64	0.6	0.360	-2.483	6.756	4.273
2010-9-6	9.0	14.1	0.294	43.59	2.1	0.353	-2.179	6.821	4.642
2010-9-7	10.5	15.3	0.337	39.30	0.3	0.340	-2.952	7.293	4.341
2010-9-8	10.1	15.0	0.398	14.55	0.4	0.323	-1.688	5.966	4.278
2010-9-9	8.9	14.7	0.352	44.59	0	0.307	-3.397	7.643	4.245
2010-9-10	9.2	15.0	0.336	42.18	0	0.291	-3.004	7.266	4.262
2010-9-11	8.9	13.7	0.217	45.98	0	0.278	-3.675	7.942	4.267
2010-9-12	10.3	14.4	0.275	39.84	0	0.301	-2.344	6.613	4.269
2010-9-13	8.6	13.4	0.273	50.58	0	0.312	-2.913	7.158	4.245
2010-9-14	8.2	12.8	0.295	50.16	0	0.307	-2.921	7.210	4.289
2010-9-15	6.5	12.8	0.286	48.31	0	0.296	-1.963	6.223	4.259
2010-9-16	6.3	12.2	0.339	48.57	0.4	0.283	-2.141	6.451	4.310
2010-9-17	6.0	9.8	0.208	45.55	0	0.273	-2.580	6.793	4.213
2010-9-18	8.6	11.9	0.249	37.20	0	0.270	-1.570	5.944	4.374
2010-9-19	8.3	13.5	0.307	43.60	0	0.265	-1.218	5.563	4.345
2010-9-20	5.9	11.7	0.178	45.82	0.3	0.256	-1.223	5.372	4.148
2010-9-21	5.0	8.0	0.111	16.74	0	0.249	-0.184	3.627	3.443
2010-9-22	6.1	11.9	0.245	28.35	0	0.246	-1.706	5.164	3.459
2010-9-23	6.0	11.2	0.279	25.35	13.4	0.242	-0.088	3.783	3.695
2010-9-24	6.3	12.1	0.243	9.75	9.3	0.236	0.124	2.985	3.108
2010-9-25	7.5	11.9	0.285	12.06	4.5	0.226	-0.009	2.598	2.589
2010-9-26	9.0	14.0	0.309	20.20	0.4	0.219	-1.082	3.720	2.637

2010-9-27	9.8	14.3	0.355	20.19	3.5	0.209	-0.650	3.485	2.835
2010-9-28	9.3	14.6	0.328	30.62	4.1	0.199	-1.242	3.912	2.670
2010-9-29	10.3	15.3	0.301	35.59	0.4	0.191	-1.486	3.975	2.489
2010-9-30	10.6	15.1	0.328	26.42	2.9	0.183	-0.832	3.598	2.765
2010-10-1	13.3	16.9	0.405	20.18	5.2	0.179	-0.045	2.645	2.599
2010-10-2	11.2	16.3	0.318	39.53	0	0.174	-0.721	3.344	2.623
2010-10-3	9.9	15.1	0.346	35.44	0	0.168	-1.058	3.564	2.505
2010-10-4	8.8	11.6	0.245	29.11	0	0.164	-0.780	3.183	2.403
2010-10-5	7.2	12.3	0.204	14.37	0.3	0.161	-0.038	2.182	2.144
2010-10-6	9.6	13.4	0.200	42.74	0	0.161	-0.545	2.711	2.166
2010-10-7	6.8	8.8	0.117	40.97	0	0.201	-0.392	2.511	2.119
2010-10-8	3.7	6.1	0.122	41.95	0	0.239	-0.176	2.286	2.110
2010-10-9	5.3	8.4	0.168	34.90	8.1	0.271	-0.317	2.480	2.163
2010-10-10	6.5	9.5	0.157	22.62	4.4	0.287	0.286	1.914	2.200
2010-10-11	5.8	8.8	0.214	18.15	0	0.315	0.131	1.940	2.071
2010-10-12	3.9	8.1	0.169	18.41	0	0.321	0.654	1.550	2.204
2010-10-13	6.9	9.9	0.225	20.58	11.8	0.322	0.526	1.603	2.128
2010-10-14	3.9	7.5	0.157	33.01	0	0.337	-0.001	1.965	1.964
2010-10-15	3.4	8.6	0.238	38.47	0	0.342	0.359	1.652	2.011
2010-10-16	3.2	7.4	0.309	36.02	0	0.334	0.541	1.449	1.990
2010-10-17	3.1	7.1	0.256	28.51	0	0.326	0.310	1.658	1.968
2010-10-18	1.9	4.9	0.183	32.28	0	0.318	0.542	1.275	1.817
2010-10-19	2.9	5.8	0.263	35.60	0	0.311	0.703	1.109	1.812
2010-10-20	1.8	5.6	0.283	37.53	0.7	0.303	0.319	1.424	1.743
2010-10-21	1.5	6.2	0.289	37.52	0	0.295	0.514	1.235	1.750
2010-10-22	3.6	7.3	0.268	25.05	0	0.291	0.749	1.082	1.831
2010-10-23	4.3	6.7	0.267	28.70	0.5	0.331	0.760	1.128	1.888
2010-10-24	3.5	6.3	0.155	13.45	3.4	0.339	1.022	0.776	1.798
2010-10-25	6.3	7.6	0.183	11.70	2.6	0.337	0.897	0.821	1.718
2010-10-26	3.8	5.6	0.128	10.76	2.2	0.339	0.911	0.784	1.695
2010-10-27	1.5	4.9	0.215	25.31	2.6	0.365	0.774	0.909	1.682
2010-10-28	1.5	5.2	0.284	10.79	3.9	0.363	0.798	0.503	1.301
2010-10-29	2.5	5.5	0.297	31.67	2	0.357	0.378	0.769	1.147
2010-10-30	1.9	4.6	0.255	33.55	0	0.353	0.513	0.567	1.080
2010-10-31	1.8	4.8	0.275	33.00	0	0.347	0.645	0.526	1.171
2010-11-1	2.8	5.0	0.290	12.92	1.4	0.341	1.183	0.000	1.183
2010-11-2	1.3	5.5	0.252	18.12	0.9	0.335	1.091	0.000	1.091
2010-11-3	3.0	5.4	0.328	32.00	0.7	0.328	1.070	0.000	1.070
2010-11-4	3.7	6.1	0.208	23.74	0	0.325	0.939	0.000	0.939
2010-11-5	5.1	7.8	0.194	31.85	0.3	0.321	0.843	0.000	0.843
2010-11-6	4.0	6.4	0.110	25.37	0	0.322	0.822	0.000	0.822
2010-11-7	0.8	4.1	0.083	22.39	0	0.330	0.818	0.000	0.818
2010-11-8	-0.1	3.6	0.078	25.58	0	0.340	0.846	0.000	0.846
2010-11-9	0.1	4.2	0.113	30.23	0	0.347	0.877	0.000	0.877
2010-11-10	0.2	3.6	0.043	25.11	0	0.357	0.904	0.000	0.904
2010-11-11	-3.6	2.6	0.112	30.67	0	0.373	0.889	0.000	0.889
2010-11-12	-1.7	3.0	0.164	28.18	0	0.371	0.894	0.000	0.894
2010-11-13	1.2	4.4	0.258	27.70	0.5	0.369	0.866	0.000	0.866
2010-11-14	0.2	2.7	0.147	20.18	0	0.369	0.864	0.000	0.864
2010-11-15	-1.5	2.8	0.076	25.87	0	0.372	0.879	0.000	0.879
2010-11-16	-1.6	2.7	0.217	26.98	0	0.369	0.872	0.000	0.872
2010-11-17	-3.0	0.7	0.186	28.39	0	0.362	0.835	0.000	0.835
2010-11-18	-4.4	0.6	0.180	21.91	0	0.351	0.785	0.000	0.785
2010-11-19	-1.5	0.5	0.250	28.32	0	0.347	0.771	0.000	0.771

2010-11-20	-3.4	0.0	0.266	26.61	0	0.342	0.742	0.000	0.742
2010-11-21	-3.5	-0.3	0.210	28.03	0	0.337	0.739	0.000	0.739
2010-11-22	-3.0	-0.6	0.234	21.82	0	0.329	0.752	0.000	0.752
2010-11-23	-2.6	-0.9	0.277	20.99	0	0.324	0.747	0.000	0.747
2010-11-24	-6.1	-1.3	0.253	26.52	0	0.314	0.731	0.000	0.731
2010-11-25	-5.2	-1.1	0.198	25.89	0	0.302	0.710	0.000	0.710
2010-11-26	-5.4	-1.2	0.225	24.30	0	0.289	0.703	0.000	0.703
2010-11-27	-4.8	-0.9	0.194	21.97	0	0.282	0.658	0.000	0.658
2010-11-28	-6.0	-0.9	0.157	24.03	0	0.275	0.656	0.000	0.656
2010-11-29	-6.0	-1.4	0.196	24.62	0	0.260	0.646	0.000	0.646
2010-11-30	-6.7	-1.3	0.177	24.50	0	0.240	0.640	0.000	0.640
2010-12-1	-6.5	-2.4	0.257	25.93	0	0.227	0.578	0.000	0.578
2010-12-2	-9.4	-2.9	0.151	25.85	0	0.174	0.546	0.000	0.546
2010-12-3	-5.7	-1.7	0.186	25.17	0	0.154	0.564	0.000	0.564
2010-12-4	-4.9	-1.2	0.272	25.39	0	0.157	0.542	0.000	0.542
2010-12-5	-7.7	-2.7	0.163	25.36	0.3	0.148	0.590	0.000	0.590
2010-12-6	-2.6	-1.1	0.251	23.37	0	0.146	0.574	0.000	0.574
2010-12-7	-5.8	-2.2	0.243	24.65	0	0.145	0.628	0.000	0.628
2010-12-8	-6.1	-2.3	0.228	25.01	0	0.138	0.579	0.000	0.579
2010-12-9	-6.4	-2.2	0.236	25.01	0	0.136	0.577	0.000	0.577
2010-12-10	-7.1	-2.7	0.188	24.67	0	0.133	0.587	0.000	0.587
2010-12-11	-7.9	-2.6	0.180	23.29	0	0.130	0.594	0.000	0.594
2010-12-12	-5.8	-1.4	0.169	22.24	0	0.131	0.607	0.000	0.607
2010-12-13	-7.9	-2.8	0.196	24.57	0.4	0.130	0.589	0.000	0.589
2010-12-14	-8.8	-4.7	0.164	21.03	0	0.123	0.527	0.000	0.527
2010-12-15	-10.2	-4.7	0.165	14.76	0.4	0.116	0.548	0.000	0.548
2010-12-16	-9.2	-4.2	0.173	24.02	0	0.114	0.501	0.000	0.501
2010-12-17	-7.8	-4.0	0.210	22.13	0	0.113	0.464	0.000	0.464
2010-12-18	-7.6	-3.5	0.178	22.14	0	0.113	0.500	0.000	0.500
2010-12-19	-9.2	-3.6	0.131	22.70	0	0.115	0.509	0.000	0.509
2010-12-20	-9.6	-5.7	0.144	17.76	0	0.111	0.501	0.000	0.501
2010-12-21	-11.1	-6.1	0.167	21.92	0	0.106	0.462	0.000	0.462
2010-12-22	-9.2	-5.7	0.162	21.87	0	0.105	0.473	0.000	0.473
2010-12-23	-9.0	-5.3	0.201	21.38	0	0.104	0.453	0.000	0.453
2010-12-24	-8.8	-4.7	0.208	18.46	0	0.105	0.495	0.000	0.495
2010-12-25	-6.6	-3.7	0.255	23.67	0.5	0.107	0.477	0.000	0.477
2010-12-26	-11.1	-6.4	0.160	24.14	0	0.104	0.408	0.000	0.408
2010-12-27	-10.7	-6.0	0.166	24.10	0	0.101	0.393	0.000	0.393
2010-12-28	-12.6	-7.9	0.152	19.84	0	0.101	0.418	0.000	0.418
2010-12-29	-16.1	-9.0	0.122	22.39	0	0.096	0.407	0.000	0.407
2010-12-30	-12.9	-7.5	0.142	24.00	0	0.095	0.426	0.000	0.426
2010-12-31	-7.0	-4.8	0.225	21.70	0	0.099	0.425	0.000	0.425
2011-1-1	-6.4	*-3.3*	0.189	18.02	0.9	0.095	0.329	0.000	0.329
2011-1-2	-10.3	-6.1	0.116	23.81	1.3	0.097	0.336	0.000	0.336
2011-1-3	-12.9	-9.2	0.124	22.97	0	0.093	0.297	0.000	0.297
2011-1-4	-10.3	-8.9	0.157	24.52	0	0.091	0.275	0.000	0.275
2011-1-5	-9.0	-6.3	0.164	22.33	0	0.092	0.285	0.000	0.285
2011-1-6	-10.8	-5.8	0.111	22.51	0	0.093	0.290	0.000	0.290
2011-1-7	-9.4	-6.6	0.150	19.44	0	0.093	0.302	0.000	0.302
2011-1-8	-8.3	-5.0	0.186	19.89	0	0.093	0.292	0.000	0.292
2011-1-9	-10.3	-5.2	0.056	21.75	1.6	0.096	0.314	0.000	0.314
2011-1-10	-10.9	-5.6	0.086	21.95	0.5	0.096	0.341	0.000	0.341
2011-1-11	-16.2	-10.5	0.073	19.30	0	0.091	0.292	0.000	0.292
2011-1-12	-13.2	-9.0	0.109	19.46	0	0.089	0.312	0.000	0.312

2011-1-13	-11.8	-7.8	0.132	22.72	0	0.090	0.299	0.000	0.299
2011-1-14	-8.4	-6.0	0.186	23.37	0	0.091	0.320	0.000	0.320
2011-1-15	-13.8	-7.9	0.126	25.60	0	0.090	0.306	0.000	0.306
2011-1-16	-12.1	-7.0	0.111	21.54	0	0.090	0.297	0.000	0.297
2011-1-17	-13.4	-7.2	0.043	20.86	1	0.092	0.313	0.000	0.313
2011-1-18	-14.2	-7.1	0.057	24.10	0	0.090	0.293	0.000	0.293
2011-1-19	-11.7	-6.3	0.069	18.50	1.2	0.092	0.310	0.000	0.310
2011-1-20	-16.6	-9.2	0.059	24.25	0	0.090	0.297	0.000	0.297
2011-1-21	-12.5	-8.5	0.107	23.36	0	0.089	0.305	0.000	0.305
2011-1-22	-12.4	-8.3	0.118	27.30	0	0.088	0.300	0.000	0.300
2011-1-23	-10.1	-5.6	0.132	24.89	0	0.090	0.315	0.000	0.315
2011-1-24	-10.6	-6.9	0.125	24.04	0	0.090	0.327	0.000	0.327
2011-1-25	-13.3	-7.9	0.117	21.58	0	0.089	0.299	0.000	0.299
2011-1-26	-8.4	-4.8	0.156	22.89	0	0.091	0.319	0.000	0.319
2011-1-27	-9.4	-5.2	0.096	19.61	0.4	0.093	0.352	0.000	0.352
2011-1-28	-13.7	-8.6	0.113	25.49	0	0.090	0.308	0.000	0.308
2011-1-29	-13.2	-7.6	0.148	24.22	0	0.089	0.303	0.000	0.303
2011-1-30	-9.6	-5.3	0.165	24.26	0	0.091	0.315	0.000	0.315
2011-1-31	-10.8	-6.3	0.146	21.56	0	0.091	0.294	0.000	0.294
2011-2-1	-11.5	-7.1	0.172	30.48	0	0.091	0.294	0.000	0.294
2011-2-2	-10.5	-7.7	0.197	20.28	0	0.090	0.276	0.000	0.276
2011-2-3	-10.2	-7.4	0.215	26.69	0	0.089	0.263	0.000	0.263
2011-2-4	-5.6	-3.6	0.286	26.97	0	0.092	0.336	0.000	0.336
2011-2-5	-5.4	-3.7	0.268	22.81	0	0.094	0.348	0.000	0.348
2011-2-6	-4.6	-2.9	0.266	28.58	0	0.096	0.370	0.000	0.370
2011-2-7	-3.2	-1.8	0.356	22.75	0	0.098	0.405	0.000	0.405
2011-2-8	-3.1	-0.9	0.252	28.41	0	0.101	0.452	0.000	0.452
2011-2-9	-5.6	-2.5	0.235	28.15	0	0.102	0.442	0.000	0.442
2011-2-10	-6.9	-2.9	0.240	29.49	0	0.101	0.384	0.000	0.384
2011-2-11	-5.3	-1.0	0.192	30.75	0	0.103	0.412	0.000	0.412
2011-2-12	-7.3	-3.3	0.196	31.25	0	0.103	0.416	0.000	0.416
2011-2-13	-10.3	-4.9	0.203	33.63	0	0.100	0.378	0.000	0.378
2011-2-14	-9.9	-4.1	0.231	27.38	0	0.098	0.365	0.000	0.365
2011-2-15	-2.2	-0.9	0.270	21.61	0	0.102	0.414	0.000	0.414
2011-2-16	-4.0	-1.1	0.182	17.52	0.6	0.105	0.396	0.000	0.396
2011-2-17	-4.7	-1.3	0.039	13.68	3.3	0.111	0.427	0.000	0.427
2011-2-18	-4.0	-1.4	0.105	24.27	0	0.110	0.466	0.000	0.466
2011-2-19	-7.4	-3.9	0.127	25.35	0	0.106	0.421	0.000	0.421
2011-2-20	-7.6	-4.0	0.159	30.42	0	0.102	0.409	0.000	0.409
2011-2-21	-4.4	-1.7	0.257	33.61	0	0.104	0.426	0.000	0.426
2011-2-22	-5.4	-2.3	0.264	31.33	0	0.106	0.418	0.000	0.418
2011-2-23	-8.2	-2.6	0.200	37.36	0	0.102	0.406	0.000	0.406
2011-2-24	-3.9	-1.4	0.306	31.78	0	0.105	0.424	0.000	0.424
2011-2-25	-1.9	0.1	0.336	28.33	0	0.110	0.450	0.000	0.450
2011-2-26	-2.4	0.3	0.239	30.35	0	0.113	0.450	0.000	0.450
2011-2-27	-4.2	-0.3	0.189	34.82	0	0.114	0.466	0.000	0.466
2011-2-28	-2.4	1.1	0.254	33.36	0	0.118	0.514	0.000	0.514
2011-3-1	-5.4	-1.2	0.222	37.03	0	0.118	0.468	0.000	0.468
2011-3-2	-4.6	-1.0	0.264	29.38	0	0.114	0.404	0.000	0.404
2011-3-3	-4.3	1.0	0.185	36.48	0	0.117	0.415	0.000	0.415
2011-3-4	-2.2	0.3	0.348	28.59	0	0.121	0.453	0.000	0.453
2011-3-5	0.1	2.0	0.364	31.21	0	0.124	0.447	0.000	0.447
2011-3-6	-2.7	0.2	0.217	26.67	0	0.129	0.488	0.000	0.488
2011-3-7	-2.8	1.4	0.352	38.46	0	0.128	0.474	0.000	0.474

2011-3-8	-4.5	0.9	0.308	NaN	0.4	0.129	0.492	0.000	0.492
2011-3-9	-4.0	1.7	0.168	NaN	0	0.136	0.528	0.000	0.528
2011-3-10	-3.7	1.8	0.174	31.40	0	0.141	0.521	0.000	0.521
2011-3-11	-5.6	0.4	0.181	38.15	0	0.145	0.518	0.000	0.518
2011-3-12	-7.6	-0.9	0.200	31.44	0	0.138	0.489	0.000	0.489
2011-3-13	-3.6	0.1	0.185	31.28	0	0.136	0.499	0.000	0.499
2011-3-14	-5.3	-0.8	0.238	18.87	0.3	0.139	0.467	0.000	0.467
2011-3-15	-9.5	-1.6	0.175	39.19	0	0.127	0.536	0.000	0.536
2011-3-16	-7.2	-0.4	0.198	43.21	0	0.125	0.497	0.000	0.497
2011-3-17	-5.6	-0.4	0.272	33.54	0	0.124	0.494	0.000	0.494
2011-3-18	-4.6	1.0	0.178	26.49	1.1	0.129	0.507	0.000	0.507
2011-3-19	-4.4	0.5	0.102	41.26	1	0.136	0.494	0.000	0.494
2011-3-20	-6.0	-2.4	0.059	34.50	10.2	0.134	0.494	0.000	0.494
2011-3-21	-9.5	-2.5	0.064	36.80	1	0.128	0.512	0.000	0.512
2011-3-22	-9.4	-2.7	0.058	47.21	0.6	0.121	0.545	0.000	0.545
2011-3-23	-6.0	-2.5	0.079	38.81	0	0.118	0.518	0.000	0.518
2011-3-24	-3.9	-0.6	0.123	47.26	0.4	0.124	0.591	0.000	0.591
2011-3-25	-3.4	-0.2	0.155	40.65	0	0.129	0.620	0.000	0.620
2011-3-26	-2.7	0.6	0.208	43.52	0	0.136	0.602	0.000	0.602
2011-3-27	-2.7	0.0	0.244	47.71	0	0.139	0.610	0.000	0.610
2011-3-28	-2.9	0.7	0.251	43.16	0	0.136	0.611	0.000	0.611
2011-3-29	0.4	3.1	0.307	40.50	0	0.147	0.672	0.000	0.672
2011-3-30	0.9	3.1	0.182	33.86	1.6	0.171	0.683	0.000	0.683
2011-3-31	2.1	4.6	0.126	31.93	1.5	0.231	0.817	0.000	0.817
2011-4-1	0.1	1.0	0.108	39.22	5.1	0.328	0.519	0.000	0.519
2011-4-2	-1.1	1.7	0.080	24.20	1.1	0.396	0.534	0.000	0.534
2011-4-3	1.3	4.0	0.116	31.73	1.7	0.427	0.686	0.000	0.686
2011-4-4	3.9	5.8	0.184	38.11	0	0.427	0.841	0.000	0.841
2011-4-5	3.1	4.6	0.199	37.28	0.3	0.412	0.774	0.000	0.774
2011-4-6	2.0	4.6	0.130	32.82	1	0.409	0.811	0.000	0.811
2011-4-7	-2.6	1.2	0.116	25.20	0.8	0.403	0.566	0.000	0.566
2011-4-8	-2.2	3.2	0.203	34.30	0.3	0.399	0.621	0.000	0.621
2011-4-9	-0.4	3.0	0.228	36.16	0	0.391	0.684	0.000	0.684
2011-4-10	1.6	4.7	0.258	43.86	0	0.384	0.832	0.000	0.832
2011-4-11	3.9	6.1	0.271	36.85	0	0.381	0.938	0.000	0.938
2011-4-12	3.7	6.7	0.286	45.29	0	0.375	0.961	0.000	0.961
2011-4-13	3.5	7.9	0.260	52.45	0	0.369	1.028	0.000	1.028
2011-4-14	3.1	6.3	0.268	40.07	0	0.365	1.049	0.000	1.049
2011-4-15	0.2	2.5	0.146	26.09	3.1	0.363	0.710	0.000	0.710
2011-4-16	0.4	3.7	0.168	37.31	0	0.364	0.750	0.000	0.750
2011-4-17	0.7	4.6	0.202	34.17	0	0.363	0.831	0.000	0.831
2011-4-18	-0.5	3.7	0.089	33.12	2.5	0.363	-0.551	1.358	0.806
2011-4-19	1.7	7.6	0.148	46.93	0.7	0.366	-1.364	2.405	1.041
2011-4-20	5.3	7.0	0.252	35.75	5.1	0.383	0.595	0.589	1.184
2011-4-21	0.7	3.1	0.151	19.45	1.5	0.379	-0.404	1.205	0.801
2011-4-22	1.3	5.2	0.215	39.66	0	0.379	-0.346	1.292	0.946
2011-4-23	1.8	6.1	0.273	43.25	0	0.372	-0.863	1.903	1.040
2011-4-24	3.1	7.4	0.349	44.53	0	0.369	-0.604	1.754	1.150
2011-4-25	4.7	8.9	0.369	56.58	0	0.368	-1.000	2.469	1.469
2011-4-26	7.0	8.9	0.569	51.12	0	0.364	-0.644	2.030	1.386
2011-4-27	5.6	8.4	0.465	49.61	0	0.361	-0.487	1.849	1.362
2011-4-28	6.2	10.2	0.500	51.37	0	0.357	-0.257	2.066	1.809
2011-4-29	7.1	11.6	0.498	55.65	0	0.354	-0.090	2.251	2.161
2011-4-30	6.9	10.4	0.436	43.01	0	0.350	-0.252	2.238	1.986

2011-5-1	4.8	9.1	0.272	35.90	2.1	0.346	-0.085	1.474	1.389
2011-5-2	2.4	8.3	0.205	48.46	0	0.345	-0.460	1.797	1.337
2011-5-3	4.1	7.1	0.351	33.81	0	0.345	0.056	1.169	1.226
2011-5-4	4.1	8.5	0.458	59.87	0	0.344	-0.753	1.986	1.233
2011-5-5	6.2	9.2	0.517	53.70	0	0.341	-0.421	1.739	1.318
2011-5-6	8.0	12.1	0.591	62.10	0	0.339	-0.857	2.415	1.558
2011-5-7	8.7	13.4	0.546	55.69	0	0.336	-0.759	2.553	1.793
2011-5-8	10.5	16.0	0.553	60.97	0	0.332	-0.638	2.850	2.213
2011-5-9	9.2	12.7	0.302	32.57	8.6	0.327	-0.602	2.723	2.122
2011-5-10	8.7	11.8	0.418	47.21	2.7	0.361	-0.320	2.286	1.966
2011-5-11	6.5	10.1	0.248	36.54	0.3	0.358	-2.091	3.530	1.439
2011-5-12	6.0	12.6	0.295	55.16	0	0.353	-3.204	4.686	1.483
2011-5-13	4.5	6.4	0.141	13.48	6.7	0.349	-3.533	4.885	1.352
2011-5-14	4.4	8.9	0.198	43.72	1.8	0.360	-3.306	4.616	1.311
2011-5-15	2.2	6.8	0.122	28.08	5.8	0.378	-2.555	3.831	1.276
2011-5-16	4.6	10.1	0.354	64.16	0	0.384	-2.403	3.755	1.352
2011-5-17	6.7	11.0	0.532	59.19	0	0.376	-3.001	4.417	1.416
2011-5-18	8.2	11.6	0.541	48.16	0	0.369	-2.012	3.480	1.469
2011-5-19	7.8	11.6	0.413	39.20	0	0.363	-1.154	2.815	1.661
2011-5-20	6.0	10.8	0.197	33.95	3.5	0.358	-1.924	3.541	1.618
2011-5-21	-0.5	2.1	0.065	28.37	6.7	0.370	-1.244	2.679	1.436
2011-5-22	3.9	5.8	0.277	59.88	0	0.408	-1.934	3.825	1.891
2011-5-23	4.0	5.3	0.140	12.88	7.6	0.410	-0.356	1.910	1.554
2011-5-24	3.9	6.6	0.198	27.75	0.5	0.423	-1.800	3.341	1.541
2011-5-25	3.9	7.5	0.206	44.26	1.4	0.413	-2.440	4.239	1.799
2011-5-26	6.4	9.4	0.349	63.68	0	0.406	-2.352	4.535	2.183
2011-5-27	7.6	10.8	0.432	50.56	0	0.392	-1.882	4.435	2.552
2011-5-28	9.7	11.5	0.405	32.93	0	0.384	-1.375	3.888	2.513
2011-5-29	9.0	11.6	0.299	29.48	1.7	0.378	-1.346	3.867	2.521
2011-5-30	3.3	6.8	0.134	25.15	15.4	0.418	-1.125	2.918	1.793
2011-5-31	5.2	10.1	0.241	51.61	0	0.423	-2.466	4.760	2.294
2011-6-1	5.4	8.1	0.207	19.98	2.2	0.416	-0.994	3.185	2.191
2011-6-2	7.7	11.8	0.328	59.22	0	0.411	-2.268	4.791	2.522
2011-6-3	9.4	12.1	0.231	29.25	11.5	0.404	-0.614	3.349	2.735
2011-6-4	9.6	11.9	0.311	33.32	1.2	0.434	-2.073	4.707	2.634
2011-6-5	6.9	9.8	0.209	17.67	7.2	0.432	-0.573	2.978	2.405
2011-6-6	7.9	11.0	0.352	50.27	0.3	0.435	-2.515	5.125	2.610
2011-6-7	8.1	12.3	0.464	63.77	0	0.422	-2.686	5.600	2.914
2011-6-8	6.3	9.1	0.230	26.73	12.5	0.416	-0.837	3.381	2.544
2011-6-9	7.9	10.3	0.349	46.90	0	0.435	-3.482	5.957	2.476
2011-6-10	9.3	11.9	0.501	51.55	0	0.421	-2.638	6.404	3.766
2011-6-11	7.6	10.9	0.321	26.54	0.8	0.412	-2.237	5.564	3.327
2011-6-12	8.2	12.8	0.349	54.24	2.8	0.406	-2.898	7.024	4.127
2011-6-13	7.4	10.7	0.212	22.81	16.7	0.441	-1.872	5.203	3.331
2011-6-14	9.6	12.9	0.397	55.26	0	0.438	-2.608	6.897	4.289
2011-6-15	10.5	13.8	0.407	56.42	1	0.421	-3.347	8.136	4.789
2011-6-16	10.4	12.8	0.246	23.59	4.3	0.423	-1.309	5.594	4.285
2011-6-17	10.5	14.3	0.417	55.22	0	0.418	-2.473	7.414	4.941
2011-6-18	11.5	16.0	0.403	61.02	0	0.402	-2.525	8.451	5.925
2011-6-19	12.4	15.7	0.268	34.77	3.1	0.387	-0.847	6.889	6.042
2011-6-20	12.6	15.0	0.227	25.41	12.7	0.408	-0.937	6.152	5.216
2011-6-21	12.4	14.8	0.253	26.60	6.3	0.440	-2.487	7.175	4.688
2011-6-22	9.5	12.6	0.234	30.06	5.9	0.450	-2.622	6.445	3.823
2011-6-23	10.4	14.5	0.405	61.03	0	0.440	-4.239	8.993	4.754

2011-6-24	10.1	15.1	0.452	59.09	0	0.420	-3.475	9.373	5.898
2011-6-25	9.4	13.3	0.267	35.05	1.4	0.404	-4.346	8.789	4.443
2011-6-26	8.0	12.2	0.287	38.28	3.9	0.405	-3.858	7.569	3.711
2011-6-27	9.2	14.1	0.415	64.38	0	0.395	-3.722	8.717	4.995
2011-6-28	10.2	15.5	0.452	63.27	0	0.376	-4.480	10.261	5.780
2011-6-29	10.8	15.0	0.400	44.54	11.8	0.358	-3.387	8.493	5.106
2011-6-30	12.8	15.1	0.253	25.44	4.1	0.388	-2.309	6.936	4.627
2011-7-1	13.8	16.0	0.276	32.88	2.8	0.411	-3.104	8.222	5.119
2011-7-2	13.6	16.9	0.294	35.74	8.7	0.438	-2.832	8.116	5.284
2011-7-3	12.2	14.4	0.145	15.92	39	0.469	-1.563	6.280	4.717
2011-7-4	12.9	16.1	0.212	32.61	8	0.469	-2.498	7.447	4.949
2011-7-5	13.3	17.5	0.251	36.09	2.2	0.466	-3.624	8.916	5.292
2011-7-6	10.6	14.9	0.242	20.21	10.2	0.462	-1.858	6.603	4.745
2011-7-7	10.1	15.2	0.364	48.67	0	0.457	-4.724	9.478	4.754
2011-7-8	11.8	17.2	0.443	61.42	0.3	0.446	-1.314	6.675	5.361
2011-7-9	9.9	14.9	0.277	31.65	19.2	0.461	-3.243	8.054	4.811
2011-7-10	6.7	12.1	0.197	22.64	2.4	0.464	-2.690	6.577	3.887
2011-7-11	7.7	11.9	0.209	22.92	0.8	0.462	-3.314	7.192	3.878
2011-7-12	7.8	11.7	0.229	16.89	0.5	0.457	-2.180	5.906	3.726
2011-7-13	8.5	12.6	0.246	25.30	0.5	0.455	-3.098	6.875	3.776
2011-7-14	9.0	13.9	0.341	46.27	0	0.450	-4.636	8.588	3.952
2011-7-15	9.5	14.9	0.368	52.50	0.8	0.441	-5.159	9.287	4.129
2011-7-16	8.5	14.7	0.240	33.25	0.8	0.433	-2.732	6.917	4.186
2011-7-17	10.0	15.7	0.407	58.04	0	0.423	-3.925	8.254	4.329
2011-7-18	11.0	17.0	0.531	58.94	0	0.408	-4.173	8.566	4.394
2011-7-19	11.3	17.5	0.472	58.02	1.4	0.392	-3.447	7.919	4.472
2011-7-20	9.9	16.0	0.301	41.44	1.9	0.377	-3.085	7.766	4.681
2011-7-21	11.0	17.0	0.390	58.64	0	0.369	-3.992	9.447	5.454
2011-7-22	11.9	18.3	0.489	61.04	0	0.359	-3.112	9.291	6.179
2011-7-23	11.8	18.6	0.431	58.77	3.2	0.346	-2.833	9.322	6.489
2011-7-24	13.8	19.5	0.479	53.72	2.4	0.336	-2.461	10.231	7.770
2011-7-25	12.1	17.6	0.361	46.56	20.2	0.331	-2.940	8.939	5.999
2011-7-26	11.6	16.9	0.326	52.05	0	0.364	-3.820	9.850	6.030
2011-7-27	12.0	16.9	0.319	46.87	6.7	0.369	-3.620	9.449	5.829
2011-7-28	10.9	15.3	0.206	18.40	10.5	0.412	-1.279	5.873	4.594
2011-7-29	9.8	14.1	0.245	23.62	4.5	0.438	-2.834	6.580	3.746
2011-7-30	9.0	13.7	0.184	27.13	0	0.435	-1.946	6.517	4.572
2011-7-31	9.7	14.9	0.389	32.85	0.9	0.430	-2.074	6.947	4.873
2011-8-1	8.3	14.8	0.280	53.46	0	0.421	-3.352	8.105	4.753
2011-8-2	8.4	14.0	0.264	37.18	0	0.410	-3.334	7.852	4.517
2011-8-3	8.6	13.2	0.210	13.76	2.2	0.403	-0.102	4.562	4.460
2011-8-4	10.4	15.4	0.442	40.94	0	0.403	-1.915	6.640	4.726
2011-8-5	9.5	15.3	0.365	46.32	0	0.394	-3.303	8.162	4.860
2011-8-6	8.6	15.3	0.344	51.50	0	0.380	-3.230	7.743	4.512
2011-8-7	8.1	14.4	0.324	44.24	1.1	0.367	-3.069	7.577	4.508
2011-8-8	9.2	15.0	0.365	53.53	0	0.355	-3.672	8.230	4.558
2011-8-9	9.7	16.1	0.368	53.98	0	0.344	-3.070	8.102	5.032
2011-8-10	11.0	16.5	0.373	52.52	0	0.331	-2.786	7.958	5.172
2011-8-11	11.8	17.2	0.411	52.18	0.3	0.320	-3.649	9.106	5.457
2011-8-12	12.8	18.2	0.426	54.14	0	0.309	-2.749	8.547	5.798
2011-8-13	13.7	18.8	0.410	53.82	0	0.298	-2.570	8.689	6.120
2011-8-14	14.1	18.9	0.356	52.48	0	0.285	-1.911	8.049	6.138
2011-8-15	13.8	19.1	0.363	49.50	4.8	0.273	0.074	6.245	6.319
2011-8-16	13.2	17.7	0.284	34.05	7	0.266	0.545	5.665	6.210

2011-8-17	12.8	17.1	0.296	45.69	1.2	0.263	-1.611	7.724	6.113
2011-8-18	13.0	16.7	0.302	39.07	46.2	0.268	-0.384	6.305	5.921
2011-8-19	12.2	14.6	0.240	35.60	30.6	0.408	-2.947	5.943	2.996
2011-8-20	12.4	15.4	0.241	24.62	11.3	0.434	-2.197	5.742	3.545
2011-8-21	12.9	16.7	0.415	44.18	6	0.432	-2.651	6.552	3.901
2011-8-22	8.3	14.3	0.210	23.92	0	0.449	-2.359	5.998	3.639
2011-8-23	7.9	13.8	0.281	48.04	0.9	0.440	-3.430	6.922	3.493
2011-8-24	7.1	13.7	0.270	46.84	0	0.430	-2.947	6.544	3.597
2011-8-25	6.4	13.0	0.260	44.67	0	0.421	-2.817	6.273	3.456
2011-8-26	7.2	13.7	0.322	52.80	0	0.411	-2.791	6.288	3.497
2011-8-27	6.9	13.4	0.304	43.91	0	0.398	-1.948	5.375	3.427
2011-8-28	8.4	14.4	0.326	53.70	0	0.386	-3.167	6.656	3.489
2011-8-29	9.3	15.0	0.367	52.95	0	0.372	-2.294	6.181	3.887
2011-8-30	10.6	15.6	0.467	52.48	0	0.358	-1.707	5.816	4.109
2011-8-31	11.4	16.3	0.458	50.91	0	0.345	-1.661	6.024	4.363
2011-9-1	13.0	17.7	0.403	47.59	0	0.334	-1.261	6.158	4.896
2011-9-2	12.7	16.7	0.258	25.76	6.8	0.325	0.840	4.273	5.114
2011-9-3	12.3	15.9	0.219	21.68	1.8	0.326	0.727	3.961	4.689
2011-9-4	13.5	16.4	0.274	31.13	2.3	0.336	0.096	4.824	4.920
2011-9-5	12.3	16.3	0.279	36.73	28.4	0.371	-0.597	5.419	4.822
2011-9-6	10.3	14.4	0.212	20.22	9.2	0.437	-1.432	5.517	4.085
2011-9-7	7.4	13.5	0.192	26.79	3.4	0.438	-1.629	5.446	3.817
2011-9-8	6.9	13.4	0.226	39.57	3.6	0.434	-2.135	5.631	3.495
2011-9-9	6.1	12.7	0.222	43.21	0	0.433	-3.110	6.228	3.118
2011-9-10	9.1	13.7	0.285	38.09	7.8	0.425	-1.907	5.229	3.321
2011-9-11	7.6	13.0	0.221	27.51	15.6	0.451	-1.902	5.004	3.102
2011-9-12	6.8	12.3	0.166	32.62	6.5	0.442	-2.548	5.509	2.961
2011-9-13	7.2	12.7	0.269	37.14	1.4	0.449	-2.705	5.806	3.101
2011-9-14	8.6	13.5	0.309	45.51	0	0.438	-2.694	5.922	3.228
2011-9-15	8.1	13.0	0.278	37.55	1.1	0.430	-1.833	4.905	3.072
2011-9-16	10.1	14.2	0.218	31.04	4.2	0.431	-0.965	4.319	3.354
2011-9-17	6.9	12.1	0.133	8.13	4.8	0.437	0.966	1.929	2.895
2011-9-18	6.8	11.7	0.209	24.16	4.2	0.448	-1.344	4.163	2.818
2011-9-19	6.2	11.3	0.125	17.48	3	0.447	-0.639	3.271	2.633
2011-9-20	7.5	12.0	0.191	28.95	0.8	0.445	-1.688	4.609	2.921
2011-9-21	7.5	11.6	0.151	19.92	0	0.440	-1.065	3.811	2.746
2011-9-22	8.0	12.4	0.199	23.35	0	0.434	-1.407	4.413	3.006
2011-9-23	7.9	12.4	0.222	28.09	0.3	0.428	-1.255	4.278	3.023
2011-9-24	7.2	12.6	0.188	27.70	5.2	0.431	-0.816	3.844	3.028
2011-9-25	6.5	11.1	0.182	14.77	0	0.432	-0.155	2.929	2.774
2011-9-26	7.4	11.5	0.196	29.30	0	0.429	-1.924	4.662	2.738
2011-9-27	7.2	10.6	0.171	13.24	10.3	0.440	-0.481	2.948	2.466
2011-9-28	3.5	9.6	0.177	16.85	3.3	0.450	-0.581	2.989	2.408
2011-9-29	4.5	10.2	0.233	34.30	0	0.443	-1.748	4.239	2.491
2011-9-30	5.4	10.3	0.238	20.26	0.9	0.437	-0.812	3.415	2.603
2011-10-1	2.5	7.9	0.091	6.32	5.6	0.439	0.460	1.676	2.136
2011-10-2	3.5	8.4	0.197	25.87	0	0.444	-1.721	3.678	1.957
2011-10-3	2.3	7.8	0.119	17.56	1.8	0.440	-0.894	2.905	2.011
2011-10-4	3.2	8.5	0.268	38.59	0	0.438	-1.408	3.426	2.018
2011-10-5	3.2	8.1	0.322	39.26	0	0.429	-1.000	2.883	1.883
2011-10-6	4.3	9.1	0.361	41.70	0	0.424	-0.793	2.882	2.089
2011-10-7	5.0	8.8	0.358	40.33	0	0.413	-0.701	2.721	2.020
2011-10-8	6.8	10.2	0.338	33.67	0	0.404	-0.158	2.370	2.212
2011-10-9	7.1	11.2	0.230	33.03	10.4	0.419	-0.392	2.795	2.403

2011-10-10	5.2	10.0	0.156	29.54	2.5	0.431	-0.432	2.816	2.384
2011-10-11	5.9	9.9	0.307	32.46	0	0.430	-0.240	2.627	2.388
2011-10-12	1.8	7.9	0.170	27.47	0.3	0.422	-0.477	2.507	2.030
2011-10-13	0.8	6.4	0.376	39.03	0	0.415	-0.458	2.214	1.756
2011-10-14	-0.4	5.1	0.310	39.01	0.3	0.407	-0.403	1.878	1.475
2011-10-15	-2.0	3.9	0.354	19.10	0	0.398	0.043	1.263	1.306
2011-10-16	-1.0	4.9	0.226	37.96	0	0.395	-0.209	1.525	1.316
2011-10-17	1.5	5.7	0.266	33.52	0	0.390	-0.140	1.506	1.365
2011-10-18	3.5	7.3	0.208	30.64	0.9	0.386	0.443	1.197	1.640
2011-10-19	1.9	6.2	0.097	23.44	9	0.402	0.306	1.192	1.499
2011-10-20	3.5	7.9	0.210	37.05	0	0.424	0.419	1.605	2.024
2011-10-21	3.7	7.4	0.236	34.08	0.5	0.417	0.146	1.818	1.965
2011-10-22	4.6	7.9	0.134	17.68	3.3	0.415	0.619	1.458	2.076
2011-10-23	2.9	7.8	0.224	27.95	0.8	0.423	0.399	1.663	2.061
2011-10-24	0.5	6.4	0.136	28.47	0	0.418	0.039	1.618	1.657
2011-10-25	1.9	5.8	0.211	29.14	0	0.412	-0.025	1.464	1.439
2011-10-26	2.4	6.9	0.139	21.70	2.8	0.410	0.539	1.107	1.646
2011-10-27	-1.8	5.3	0.068	20.95	1.8	0.414	-0.246	1.523	1.277
2011-10-28	-3.1	4.4	0.110	33.80	0	0.419	-0.596	1.463	0.867
2011-10-29	-2.4	3.9	0.156	35.86	0	0.414	0.815	0.000	0.815
2011-10-30	-0.6	3.9	0.175	32.39	0	0.410	0.786	0.000	0.786
2011-10-31	0.5	4.6	0.221	34.74	0	0.407	0.890	0.000	0.890
2011-11-1	0.4	4.5	0.163	24.02	0	0.403	0.758	0.000	0.758
2011-11-2	0.0	4.7	0.077	12.32	0.4	0.403	0.745	0.000	0.745
2011-11-3	-0.6	4.4	0.109	21.34	0	0.402	0.736	0.000	0.736
2011-11-4	-1.5	3.4	0.087	20.09	0	0.398	0.686	0.000	0.686
2011-11-5	-1.0	3.8	0.120	26.33	0	0.397	0.737	0.000	0.737
2011-11-6	-0.7	3.0	0.082	12.25	3.4	0.394	0.771	0.000	0.771
2011-11-7	-3.5	2.3	0.126	28.75	3	0.399	0.699	0.000	0.699
2011-11-8	-6.2	1.0	0.132	32.08	0	0.407	0.726	0.000	0.726
2011-11-9	-5.3	0.2	0.185	34.47	0	0.402	0.740	0.000	0.740
2011-11-10	-3.9	0.2	0.161	32.52	0	0.395	0.743	0.000	0.743
2011-11-11	-0.1	1.6	0.213	28.87	0	0.397	0.658	0.000	0.658
2011-11-12	-1.7	1.8	0.092	16.79	2.6	0.408	0.662	0.000	0.662
2011-11-13	-4.2	1.6	0.058	19.51	0	0.410	0.689	0.000	0.689
2011-11-14	-1.8	1.8	0.113	21.60	0.6	0.409	0.722	0.000	0.722
2011-11-15	-1.8	2.0	0.095	18.66	0	0.410	0.695	0.000	0.695
2011-11-16	-2.1	1.4	0.132	27.69	1.2	0.408	0.683	0.000	0.683
2011-11-17	-2.3	1.1	0.146	24.89	0	0.405	0.709	0.000	0.709
2011-11-18	-3.9	0.4	0.178	29.77	0	0.399	0.700	0.000	0.700
2011-11-19	-3.8	-0.1	0.221	29.94	0	0.386	0.713	0.000	0.713
2011-11-20	-1.9	-0.3	0.229	29.14	0	0.374	0.667	0.000	0.667
2011-11-21	-3.8	-0.2	0.151	21.66	0	0.372	0.688	0.000	0.688
2011-11-22	-2.5	-0.3	0.201	27.22	0	0.367	0.652	0.000	0.652
2011-11-23	-3.1	-0.4	0.223	26.59	0	0.358	0.707	0.000	0.707
2011-11-24	-2.5	-0.4	0.215	28.08	0	0.353	0.665	0.000	0.665
2011-11-25	-1.7	-0.3	0.243	28.49	0	0.352	0.662	0.000	0.662
2011-11-26	-1.5	-0.2	0.251	28.95	0	0.353	0.697	0.000	0.697
2011-11-27	-3.2	-0.3	0.212	26.30	0	0.350	0.737	0.000	0.737
2011-11-28	-2.4	-0.3	0.207	22.65	0	0.347	0.689	0.000	0.689
2011-11-29	-1.9	-0.2	0.326	21.52	0	0.349	0.620	0.000	0.620
2011-11-30	-5.9	-0.4	0.123	16.03	0	0.343	0.680	0.000	0.680
2011-12-1	-6.7	-0.8	0.221	25.90	0	0.329	0.589	0.000	0.589
2011-12-2	-6.8	-1.4	0.186	24.34	0	0.292	0.564	0.000	0.564

2011-12-3	-3.8	-1.0	0.217	21.26	0	0.266	0.634	0.000	0.634
2011-12-4	-4.9	-0.8	0.154	13.44	0	0.253	0.631	0.000	0.631
2011-12-5	-4.3	-0.7	0.144	23.84	0	0.247	0.643	0.000	0.643
2011-12-6	-4.5	-0.5	0.114	13.86	0	0.250	0.632	0.000	0.632
2011-12-7	-6.9	-0.8	0.079	17.70	0	0.241	0.549	0.000	0.549
2011-12-8	-7.4	-1.0	0.076	18.57	0	0.221	0.540	0.000	0.540
2011-12-9	-8.3	-1.3	0.097	20.77	0	0.194	0.519	0.000	0.519
2011-12-10	-10.3	-1.6	0.102	17.60	0	0.168	0.479	0.000	0.479
2011-12-11	-6.6	-1.2	0.098	18.20	1	0.159	0.497	0.000	0.497
2011-12-12	-8.3	-1.7	0.117	23.14	0	0.152	0.493	0.000	0.493
2011-12-13	-8.5	-2.1	0.142	24.12	0	0.139	0.472	0.000	0.472
2011-12-14	-8.6	-1.8	0.109	20.72	0	0.137	0.452	0.000	0.452
2011-12-15	-9.7	-2.2	0.150	23.04	0	0.132	0.410	0.000	0.410
2011-12-16	-11.2	-2.6	0.085	16.26	1.2	0.126	0.378	0.000	0.378
2011-12-17	-9.6	-1.5	0.039	10.52	0.6	0.131	0.446	0.000	0.446
2011-12-18	-13.8	-3.0	0.038	16.72	0	0.123	0.449	0.000	0.449
2011-12-19	-9.2	-2.9	0.091	19.25	0	0.120	0.436	0.000	0.436
2011-12-20	-7.6	-2.5	0.127	21.38	0	0.121	0.450	0.000	0.450
2011-12-21	-10.4	-2.8	0.093	17.20	0	0.120	0.435	0.000	0.435
2011-12-22	-11.7	-3.4	0.073	20.89	0	0.116	0.430	0.000	0.430
2011-12-23	-11.4	-3.5	0.104	21.05	0	0.115	0.425	0.000	0.425
2011-12-24	-13.0	-4.0	0.101	21.73	0	0.111	0.419	0.000	0.419
2011-12-25	-9.5	-3.5	0.121	21.14	0	0.112	0.410	0.000	0.410
2011-12-26	-4.4	-2.5	0.225	20.33	0	0.115	0.445	0.000	0.445
2011-12-27	-4.1	-1.6	0.255	17.07	0	0.121	0.429	0.000	0.429
2011-12-28	-6.5	-2.0	0.119	19.65	0	0.120	0.449	0.000	0.449
2011-12-29	-6.7	-2.1	0.149	22.23	0	0.119	0.434	0.000	0.434
2011-12-30	-9.0	-2.8	0.166	22.54	0	0.116	0.453	0.000	0.453
2011-12-31	-10.5	-3.4	0.141	22.90	0	0.113	0.422	0.000	0.422
